# BdCIPK31, a Calcineurin B-Like Protein-Interacting Protein Kinase, Regulates Plant Response to Drought and Salt Stress

**DOI:** 10.3389/fpls.2017.01184

**Published:** 2017-07-07

**Authors:** Qingchen Luo, Qiuhui Wei, Ruibin Wang, Yang Zhang, Fan Zhang, Yuan He, Shiyi Zhou, Jialu Feng, Guangxiao Yang, Guangyuan He

**Affiliations:** ^1^The Genetic Engineering International Cooperation Base of Chinese Ministry of Science and Technology, Key Laboratory of Molecular Biophysics of Chinese Ministry of Education, College of Life Science and Technology, Huazhong University of Science and TechnologyWuhan, China; ^2^Hubei University of EducationWuhan, China

**Keywords:** ABA, *Brachypodium distachyon*, CIPK, drought stress, *Nicotiana tabacum*, salt stress

## Abstract

Calcineurin B-like protein interacting protein kinases (CIPKs) are vital elements in plant abiotic stress signaling pathways. However, the functional mechanism of CIPKs has not been understood clearly, especially in *Brachypodium distachyon*, a new monocot model plant. In this study, *BdCIPK31,* a *CIPK* gene from *B. distachyon* was characterized. *BdCIPK31* was downregulated by polyethylene glycol, NaCl, H_2_O_2_, and abscisic acid (ABA) treatments. Transgenic tobacco plants overexpressing *BdCIPK31* presented improved drought and salt tolerance, and displayed hypersensitive response to exogenous ABA. Further investigations revealed that BdCIPK31 functioned positively in ABA-mediated stomatal closure, and transgenic tobacco exhibited reduced water loss under dehydration conditions compared with the controls. BdCIPK31 also affected Na^+^/K^+^ homeostasis and root K^+^ loss, which contributed to maintain intracellular ion homeostasis under salt conditions. Moreover, the reactive oxygen species scavenging system and osmolyte accumulation were enhanced by *BdCIPK31* overexpression, which were conducive for alleviating oxidative and osmotic damages. Additionally, overexpression of *BdCIPK31* could elevate several stress-associated gene expressions under stress conditions. In conclusion, BdCIPK31 functions positively to drought and salt stress through ABA signaling pathway. Overexpressing *BdCIPK31* functions in stomatal closure, ion homeostasis, ROS scavenging, osmolyte biosynthesis, and transcriptional regulation of stress-related genes.

## Introduction

Abiotic stress severely constrains the growth and development of plants, causes crop yield losses and even death (Bartels and Sunkar, 2005). Plants have developed a complex mechanism to prevent damages caused by environmental changes. ABA is a pivotal element in this mechanism. ABA functions in seed germination inhibition, growth regulation, fruit abscission, and stomatal closure (Raghavendra et al., 2010). Ca^2+^ serves as a second messenger in ABA signaling pathway (Luan, 2009). The fluctuation of cytoplasmic calcium concentration ([Ca^2+^]_cyt_) initiates a series of intracellular biochemical and physiological changes (Luan, 2009). Ca^2+^ sensors detect the change in [Ca^2+^]_cyt_ and transduce the signal to sensor responder proteins (Yu et al., 2014). During the past 20 years, multiple families of plant Ca^2+^ sensors and responders are identified, including calmodulins, CDPKs, CBLs, and CIPKs (Asano et al., 2005; Luan, 2009). Among these proteins, CBL-CIPK networks and CDPKs are unique to higher plants (Liu and Zhu, 1998; Kudla et al., 1999).

The typical structure of the CIPK protein contains a kinase activation domain, a regulatory domain, and a junction domain connecting the two domains (Batistic and Kudla, 2004). In the CBL-CIPK network, Ca^2+^-bound CBL interacts with the CIPK NAF/FISL motif and activates CIPK. Afterward, the CIPK can phosphorylate its targets and transduce the Ca^2+^ signal downstream (Luan, 2009). Genome-wide analyses have suggested 26 *CIPKs* in *Arabidopsis* (Kolukisaoglu et al., 2004), 33 *CIPKs* in rice (Kolukisaoglu et al., 2004; Piao et al., 2010), 43 *CIPKs* in maize (Chen et al., 2011), and 23 *CIPKs* in canola (Zhang et al., 2014).

A few studies have indicated that the CIPKs function in plant development (Tripathi et al., 2009; Held et al., 2011), nutrient uptake (Li et al., 2006; Xu et al., 2006; Cheong et al., 2007; Chen et al., 2012), and pollen tube elongation (Zhou et al., 2015). However, the CIPKs perform major functions in stress responses. The first characterized CBL-CIPK network is the SOS network. Plasma membrane-localized SOS3 (AtCBL4) recruits SOS2 (AtCIPK24). Subsequently, the CBL-CIPK complex activates a Na^+^/H^+^ antiporter SOS1 (AtNHX7), enhances sodium export, and promotes salt tolerance (Liu and Zhu, 1997, 1998; Qiu et al., 2002). The *atcipk3* mutant shows a hypersensitive phenotype when treated with salt and ABA (Kim et al., 2003). By contrast, the *atcipk1* mutant is hypersensitive to osmotic treatment, but insensitive to ABA (D’Angelo et al., 2006). The *atcipk23* mutant exhibits enhanced drought tolerance by regulating leaf transpiration (Cheong et al., 2007), whereas the *atcipk21* mutant presents impaired salt and osmotic tolerance (Pandey et al., 2015). Similar CBL-CIPK networks are also found in other plant species including wheat. TaCIPK14 and TaCIPK29, which enhance salt and low temperature resistance in transgenic tobacco, respectively (Deng et al., 2013a,b), whereas TaCIPK25 negatively regulated salt tolerance in transgenic wheat (Jin et al., 2016). The overexpression of the constitutively activated form of *BnCIPK6* enhances salt and low-K^+^ tolerances, as well as ABA sensitivity in *Arabidopsis* (Chen et al., 2012). The MdCIPK6L improves tolerance to salt, drought, and chilling stress in transgenic apples and *Arabidopsis* (Wang et al., 2012). Notably, ABA participates extensively in the responses of *CIPK* genes to abiotic stress. Among the 21 stress-responsive *OsCIPK* genes, 17 *OsCIPKs* are also responsive to ABA (Xiang et al., 2007).

*Brachypodium distachyon*, belonging to the *Pooideae* subfamily, has close evolutionary relationship with rice, wheat, and sorghum (Vogel et al., 2010). Therefore, the characterization of *B. distachyon* genes could provide reference for studies of other species in *Pooideae* subfamily. Our previous study identified 32 *CIPK* (*SnRK3*) genes in the *Brachypodium* genome (Wang et al., 2015). However, none of these *BdCIPKs* has been functionally characterized. In the current study, we characterized a *BdCIPK* gene, designated *BdCIPK31*. Transgenic tobacco overexpressing *BdCIPK31* exhibited tolerance to drought and salt stress and hypersensitivity to ABA. These findings indicated that BdCIPK31 participates in plant’s response to abiotic stress and ABA signaling.

## Materials and Methods

### Plant Materials Preparation

*Brachypodium distachyon* inbred line Bd21 was employed as plant material. Sterilized seeds were germinated in Petri dishes with water at 25°C. For stress and signal-molecule treatments, 10-day-old seedlings were treated with 20% (w/v) PEG6000, 200 mM NaCl, 10 mM H_2_O_2_ or 100 μM ABA solutions. Normal growing seedlings were employed as untreated control. The leaves of these seedlings were harvested in time for total RNA extraction. For organ-specific expression analysis, leaves, stems, roots, and spikelets were collected from 6-week-old mature *Brachypodium* plants grown in soil. All the seedlings were cultured in 25°C, 14 h light/10 h dark cycle.

### Cloning and Bioinformatics Analysis of the *BdCIPK31* Gene

The cDNA of the *BdCIPK31* gene (accession No. XM_003562376) was amplified by primer P1 (Supplementary Table S1) using mRNA extracted from 10-day-old *Brachypodium* seedlings. Homologs of BdCIPK31 were searched using NCBI BlastP^1^ and aligned by ClustalX. The phylogenetic analysis was performed by MEGA 6.0 using the neighbor-joining method.

### Yeast-Two-Hybrid Assay

The CDS of *BdCIPK31* gene was introduced into p*GADT7* while the *BdCBLs* were introduced into p*GBKT7* (The primers are shown in Supplementary Table S1). Subsequently, The *AD-BdCIPK31* and the *BD-BdCBLs* constructs were co-transformed into yeast strain AH109 by using Yeastmaker^TM^ Yeast Transformation System 2 (Clontech, CA, United States). The transformants grown on DDO/-Leu/-Trp were diluted and dotted on TDO/-Leu/-Trp/-His for screening.

### Subcellular Localization of BdCIPK31 Protein

The CDS of the *BdCIPK31* gene was introduced into p*CAMBIA1303* using *Bgl*II and *Spe*I restriction enzyme sites. The recombinant plasmid was transformed into tobacco leaf epidermis via *Agrobacterium*-mediated transformation as described (Sparkes et al., 2006). A fluorescence microscope (Olympus IX71, Japan) was used to detect the fluorescence.

### qRT-PCR and Semi-Quantitative PCR Analysis

RNA samples were prepared with the RNAprep Pure Plant Kit (TIANGEN, Beijing, China), and cDNA was prepared with the FastQuant RT Kit (TIANGEN, Beijing, China). The semi-quantitative PCR was used to detect the expression of *BdCIPK31* in different transgenic tobacco lines. The *Nicotiana tabacum ubiquitin* gene was employed as endogenous control. The qRT-PCR analysis was applied with the SYBR Green PreMix (TIANGEN, Beijing, China) and the CFX96^TM^ Real-Time Detection System (Bio-Rad, United States). The *Brachypodium β-actin* gene and the *N. tabacum ubiquitin* gene were employed as endogenous controls. All primers applied in these assays are listed in Supplementary Table S2. The 2^-ΔΔCt^ method was used to analyze the qRT-PCR results.

### Promoter Analysis and GUS Staining

A 2-kb fragment of the *BdCIPK31* flanking 5′-upstream region before the translation initiation site (ATG) was identified in the Ensembl Plants database^2^ and amplified with primers containing the *Eco*RI and *Bam*HI restriction sites (Primer P3, Supplementary Table S1). The *cis* elements were analyzed by the Plant-CARE^3^ database. The promoter fragment was introduced into the p*BI121-GUS* vector, thereby replacing the *CaMV* 35S promoter to recombine with the Pro*_BdCIPK31_*::*GUS* construct. The construct and control p*BI121-GUS* were transformed into the *Agrobacterium tumefaciens* strain EHA105. Transgenic tobacco lines were generated by *Agrobacterium*-mediated transformation (Horsch et al., 1985). The transgenic seedlings were treated on 1/2 MS media supplied with 300 mM mannitol, 200 mM NaCl, or 5 μM ABA, respectively, for 24 h. For histochemical staining assay, the seedlings were infiltrated in GUS staining solutions (0.5 mg/mL 5-bromo-4-chloro-3-indolyl glucuronide, 0.1 M Na_2_HPO_4_, 10 mM Na_2_EDTA, 0.5 mM K_4_Fe(CN)_6_, and 0.06% Triton X-100, pH 7.0), vacuumized for 10 s, and placed at 37°C for 24 h. The stained seedlings were decolorized using ethanol. The quantitative GUS activity assay was performed by using 4-MUG method according to Jefferson et al. (1987).

### Generation of *BdCIPK31*-Overexpression Transgenic Tobacco Plants and Stress Tolerance Assays

The CDS of *BdCIPK31* gene was amplified and subcloned into p*CAMBIA1303* vector by using primer P1 (Supplementary Table S1). The p*CAMBIA1303-BdCIPK31* and vacant vector p*CAMBIA1303* were transformed into the tobacco, respectively, as described above. The transformants were selected by 20 mg/L hygromycin and confirmed by reverse transcript PCR with primer P1. Three independent T_2_ homozygote lines were used in this experiment.

For the root length and fresh weight (FW) assay, the seeds were sown on 1/2 MS medium, then transferred into media that contained 150/300 mM mannitol, 150/200 mM NaCl, 2/5 μM ABA, or 0.1 mM Tu, respectively. The root length or FW of all tested seedlings were measured before and after treatment.

For stress tolerance assay, seeds were grown on 1/2 MS for 10 days in 25°C with 16 h light/8 h dark cycle, and then were transplanted into pots containing well-watered soil, each pot contains six tobacco seedlings. For normal growth control, all the tobacco plants were well-watered at 28°C with 16 h light/8 h dark cycle. For drought treatment, 4-week-old plants were grown without watering for 35 days. The survival rate was calculated after recovery for 7 days. To examine the water loss, leaves were detached and weighed at the indicated time points. Calculation of water loss rate was according to initial weight as described previously (Hu W. et al., 2013). In salt treatment, to ensure the consistency of NaCl concentration in different pots, all pots were placed in a basin containing 2 L 800 mM NaCl. The survival rate was calculated at 7 days after treatment.

### Measurement of Stomatal Aperture

Leaves were detached from normal mature seedlings and floated in a buffer solution (30 mM KCl and 10 mM MES-KOH, pH 6.15), and incubated at 25°C under light for 5 h to fully open the stomata. For drought-induced stomatal closure, the leaves were exposed to air under light for 2 h. For ABA-induced stomatal closure, the appropriate concentrations of ABA was added. For Tu treatment, 0.3 mM Tu was applied in the buffer solution, and the treated leaves were exposed to air for 2 h after 5-h-incubation. The epidermal peels were stripped from the leaves and examined under a microscope (IX71, Olympus, Japan). The width and length of stomata were measured by Olympus cellSens software (Olympus, Japan).

### Measurement of Ion Content and K^+^ Flux

Ten-day-old seedlings were treated on 1/2 MS containing 200 mM NaCl for 7 days. The seedlings was separated into the shoot and root samples. The sample pretreatment was performed according to Deng et al. (2013a). The ion content was measured by atomic absorption spectroscopy (180-50, GFAAS; HITACHI, Japan). The net K^+^ efflux was measured by the Xuyue-Science & Technology Co. (Shanghai, China) via a non-injuring technique (NMT, Younger USA Science and Technology Corp., Amherst, MA, United States) as previously described (Hu Y. et al., 2013).

### Measurement of ABA Content

Four-week-old tobacco plants in soil were withheld watering for 7 days or treated by NaCl for 3 days. Fresh leaves were detached from treated plants and grinded in liquid nitrogen. Sample pretreatment was performed according to Yan et al. (2014). The ABA level was measured with an ELISA kit (Jiancheng, China).

### Measurement of RWC, Water Loss Rate, IL, and the MDA, Chlorophyll, Proline, Ascorbic Acid, Soluble Sugar, Ascorbic Acid, and Anthocyanin Contents in Leaves

Four-week-old tobacco plants in soil were withheld watering for 7 days or treated by NaCl solution for 3 days. For RWC measurement, the FW of leaves was determineded. After incubation in water overnight, the turgid weight (TW) was measured. Subsequently, the samples were dried at 70°C for 2 days and the dried weight (DW) was tested. RWC (%) = [(FW - DW)/(TW-DW)] × 100%. For water loss rate calculation, the leaves from tobacco plants under normal condition were detached and exposed to air. The initial FW (W_0_) of the leaves was recorded, and the leave weight was also measured at indicated time points after detaching as Wt. The water loss rate (%) = (W_0_ - Wt)/W_0_ × 100%. For IL measurement, the leaves from plants were cut into small pieces and immersed in 8 mL deionized water. After 12 h incubation, the conductivity (C1) was detected with a conductivity meter (DDBJ-350, Shanghai, China). Subsequently, the samples were boiled for 15 min. After cooling, the conductivity (C2) was recorded again, such that IL (%) = (C1/C2) × 100%. The MDA level was measured using the TBA reaction (Heath and Packer, 1968). The UV spectrophotometry technique was used to measure the chlorophyll content (Yang et al., 2009). The proline and ascorbic acid contents in leaves were measured with the corresponding detection kits (Jiancheng, China). The level of soluble sugar in leaves was examined as described (Kong et al., 2011). The analysis of the relative anthocyanin content was done according to previous described method (Cui et al., 2014).

### Measurement of Enzyme Activity

The activities of CAT, POD, SOD, and GST in leaves from tested plants were examined with the corresponding activity detection kit (Jiancheng, China) using leaves detached from the treated plants.

### Expression Analysis of the Ion-Transporter Genes, ROS-Scavenging System Genes, Osmolyte Synthesis Genes, and Stress-Related Genes

Four-week-old tobacco plants in soil were withheld watering for 7 days or treated by NaCl solution for 3 days. The leaves from drought treated plants were detached and subjected to RNA extraction, and these RNA samples were used to analyze the *TORK1* transcripts, as well as ROS-scavenging system genes, osmolyte synthesis genes, and stress-responsive genes. Two-week-old young seedlings were grown on 1/2 MS containing 200 mM NaCl for 2 days, and the samples were used for expression analyses of *NKT1*, *NKT2*, *NtKC1*, *NtNHX2*, and *NtNHX4,* respectively.

### Statistical Analysis

The SPSS software (Chicago, IL, United States) was used to analyze the data. The data variance analysis was performed by the ANOVA Duncan’s test.

## Results

### Characterization of BdCIPK31

The cDNA of a *CIPK* gene was amplified by RT-PCR using mRNA isolated from *B. distachyon* seedlings. The coding sequence is 1,347 bp in length, which encodes a 448-amino-acid protein. Phylogenic analysis of the predicted BdCIPK and 33 OsCIPKs showed that the BdCIPK was clustered to the exon-rich group (group A), and exhibited closest relationship with OsCIPK31 and OsCIPK3 (Supplementary Figure S1). Since this BdCIPK shared highest identity with OsCIPK31 (82.2%), the BdCIPK was designated as BdCIPK31 (XM_003562376).

The BdCIPK31 presented high identities with AtCIPK3 (70%), OsCIPK31 (82%), and TaCIPK3 (89%). Multiple sequence alignment with these homologs indicated that BdCIPK31 contained all the typical features of CIPK domains, namely, an activation loop, NAF/FISL motif, and a protein–phosphatase interaction domain (Supplementary Figure S2).

**FIGURE 1:**
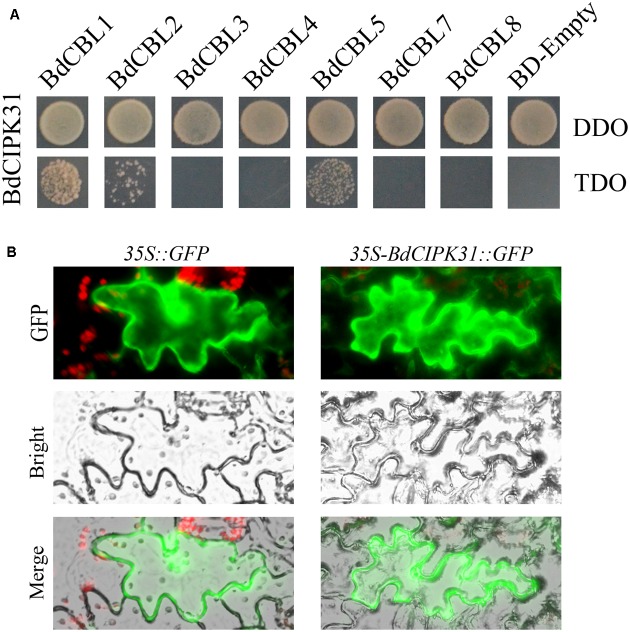
BdCIPK31-BdCBLs interaction and BdCIPK31 subcellular localization analysis. **(A)** Yeast-two-hybrid analysis of the interactions between BdCIPK31 and BdCBLs. The p*GADT7-BdCIPK31* and p*GBKT7-BdCBLs* vectors were co-transformed into AH109. The transformants were screened by DDO/-Leu/-Trp and TDO/-Leu/-Trp/-His for 3 days. **(B)** Subcellular localization analysis of BdCIPK31::GFP. The left column shows the epidermal cells expressing the free GFP protein as controls. The right column shows the epidermal cells expressing the BdCIPK31::GFP fusion protein. Three independent biological replicates were performed and produced similar results.

The yeast-two-hybrid assay was performed to identify the BdCIPK31 interacting BdCBLs, the results showed that the BdCIPK31 interacted with BdCBL1 (XP_003559049), BdCBL2 (XP_003574350), and BdCBL5 (XP_003578877) (**Figure 1A**).

To determine the subcellular localization of BdCIPK31, a recombinant vector was constructed to fuse the BdCIPK31 with fluorescent protein GFP. The constructed plasmid was transformed into tobacco leaves. The transformed epidermis was examined after 72 h. The fluorescence of BdCIPK31::GFP fusion protein was observed throughout the cell, similar to that of the green fluorescent protein used as control (**Figure 1B**).

### *BdCIPK31* Expression Responds to Abiotic Stress and Exogenous ABA

**FIGURE 2:**
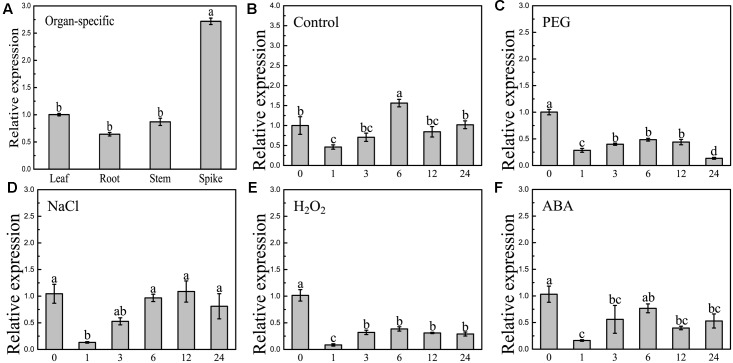
Expression patterns analyses of *BdCIPK31* gene. **(A)** Organ-specific expressions of *BdCIPK31* in leaf, root, stem, and spike of 6-week-old plant. Ten-day-old seedlings were treated by: **(B)** no treatment control, **(C)** 20% PEG6000, **(D)** 200 mM NaCl, **(E)** 100 mM H_2_O_2_, and **(F)** 100 μM ABA treatment, respectively. The *BdCIPK31* expression in the leaves of treated seedlings was detected. Data in this figure represent the mean ± SE of three independent replicates. Different letters represent significant difference in each condition (Duncan’s test, *P* < 0.05).

Quantitative reverse-transcription PCR was employed to examine the expression patterns of *BdCIPK31*. Organ-specific expression analysis showed that *BdCIPK31* was expressed in all *B. distachyon* organs tested (**Figure 2A**). Young seedlings were treated with osmotic stress generator PEG6000, salt stress generator NaCl, oxidative stress inducer H_2_O_2_, and stress-related phytohormone ABA. As shown in **Figures 2B–F**, *BdCIPK31* expression was significantly suppressed 1 h after all treatments. After 6 h of treatment, the expression levels raised again after a fall (0.5-fold in PEG, 1-fold in NaCl, 0.4-fold in H_2_O_2_, and 0.76-fold in ABA) and then downregulated after a longer period (>6 h) of exposure to all treatments. In general, the results showed that the *BdCIPK31* expression was downregulated by PEG6000, NaCl, H_2_O_2_, and ABA (Supplementary Figure S10).

### Promoter Analysis of *BdCIPK31*

To investigate the transcriptional regulation mechanism of *BdCIPK31*, we obtained a 2 kb fragment from the 5′-flanking region of the transcriptional initiation site (ATG) of *BdCIPK31* from Ensembl Plants^2^ and analyzed the sequence by the PlantCARE^3^ database. Various *cis* elements associated with the response to stress and hormone, such as the ABRE, were found in this region. Notably, no dehydration response element was discovered (Supplementary Table S3). These findings suggest that the transcriptional regulation of *BdCIPK31* may be through ABA-dependent pathway.

The result was confirmed by introducing the promoter fragment into p*BI121* to generate the Pro*_BdCIPK31_*::*GUS* construct. The constructed vector was then transformed into tobacco (*N. tabacum* var. Samsun). Ten-day-old transgenic seedlings were treated with 300 mM mannitol, 200 mM NaCl, or 5 μM ABA. After 1 day treatment, the transgenic seedlings displayed lighter GUS staining than the controls (Supplementary Figure S3), and the observation was further confirmed by quantitative GUS activity assay (Supplementary Figure S4). This finding is consistent with the qRT-PCR results.

### Overexpression of *BdCIPK31* Enhances Drought and Salt Stress Tolerance in Tobacco

To characterize the function of BdCIPK31, transgenic tobacco overexpressing *BdCIPK31* under control of *CaMV 35S* promoter was generated. Five independent transgenic lines (T_1_) were obtained (Supplementary Figure S5). The transcript level of *BdCIPK31* in each T_2_ homozygous line was examined by semi-quantitative RT-PCR. Among these lines, OE2, OE4, and OE8, which expressed *BdCIPK31* at different levels, were selected as experimental materials (Supplementary Figure S6). Transgenic tobacco plants transformed with vacant p*CAMBIA1303* vectors were also generated as VCs.

**FIGURE 3:**
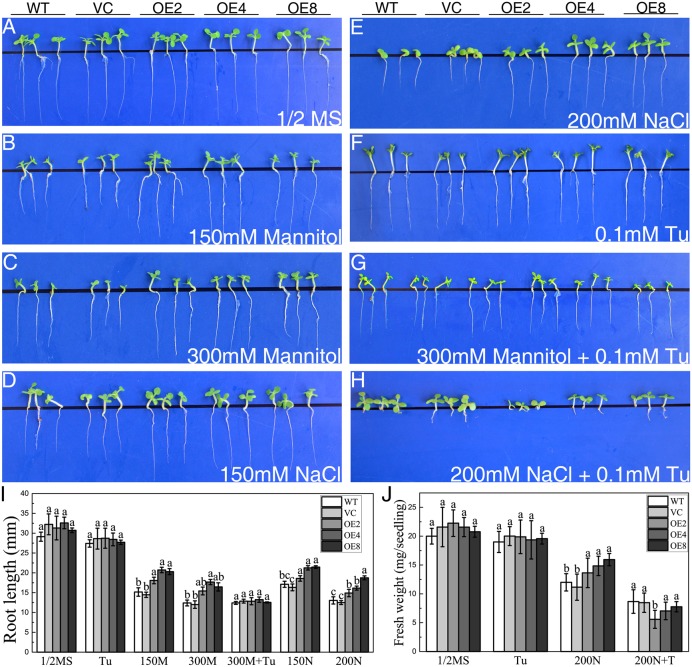
Analysis of root elongation and fresh weight in control and OE plants exposed to osmotic and salt stress. Seedlings grown on **(A)** 1/2 MS medium, **(B,C)** 1/2 MS medium containing **(B)** 150/**(C)** 300 mM mannitol, **(D,E)** 1/2 MS medium containing **(D)** 150/**(E)** 200 mM NaCl, **(F)** 1/2 MS medium containing 0.1 mM Tu, **(G)** 1/2 MS medium containing 300 mM mannitol and 0.1 mM Tu, and **(H)** 1/2 MS medium with 200 mM NaCl and 0.1 mM Tu for 14 days. **(I)** Statistical analysis of root length. **(J)** Statistical analysis of fresh weight. Data in **(I,J)** represent the mean ± SE from three independent replicates. Different letters represent significant difference in each condition (Duncan’s test, *P* < 0.05).

The responses of transgenic young seedlings to drought and salt were initially examined. The young seedlings were grown on 1/2 MS media supplied with 150/300 mM mannitol or 150/200 mM NaCl after seed germination. The seedlings of all lines displayed similar root length on the medium without treatments after 14 days (**Figure 3A**). However, OE seedlings exhibited longer root lengths than the controls under osmotic and salt stress (**Figures 3B–I**).

To elucidate whether endogenous ABA participates in the mechanism of the stress-tolerance phenotype, Tu was employed as a ABA biosynthesis inhibitor. WT, VC, and OE seedlings exhibited similar growth status on the medium containing 0.1 mM Tu (**Figure 3F**). However, the stress-tolerance phenotype of OE lines disappeared under 300 mM mannitol treatment and even reversed under 200 mM NaCl treatment by supplying with 0.1 mM Tu (**Figures 3G,H**). The results suggested that the stress tolerance regulation of BdCIPK31 was dependent on endogenous ABA.

**FIGURE 4:**
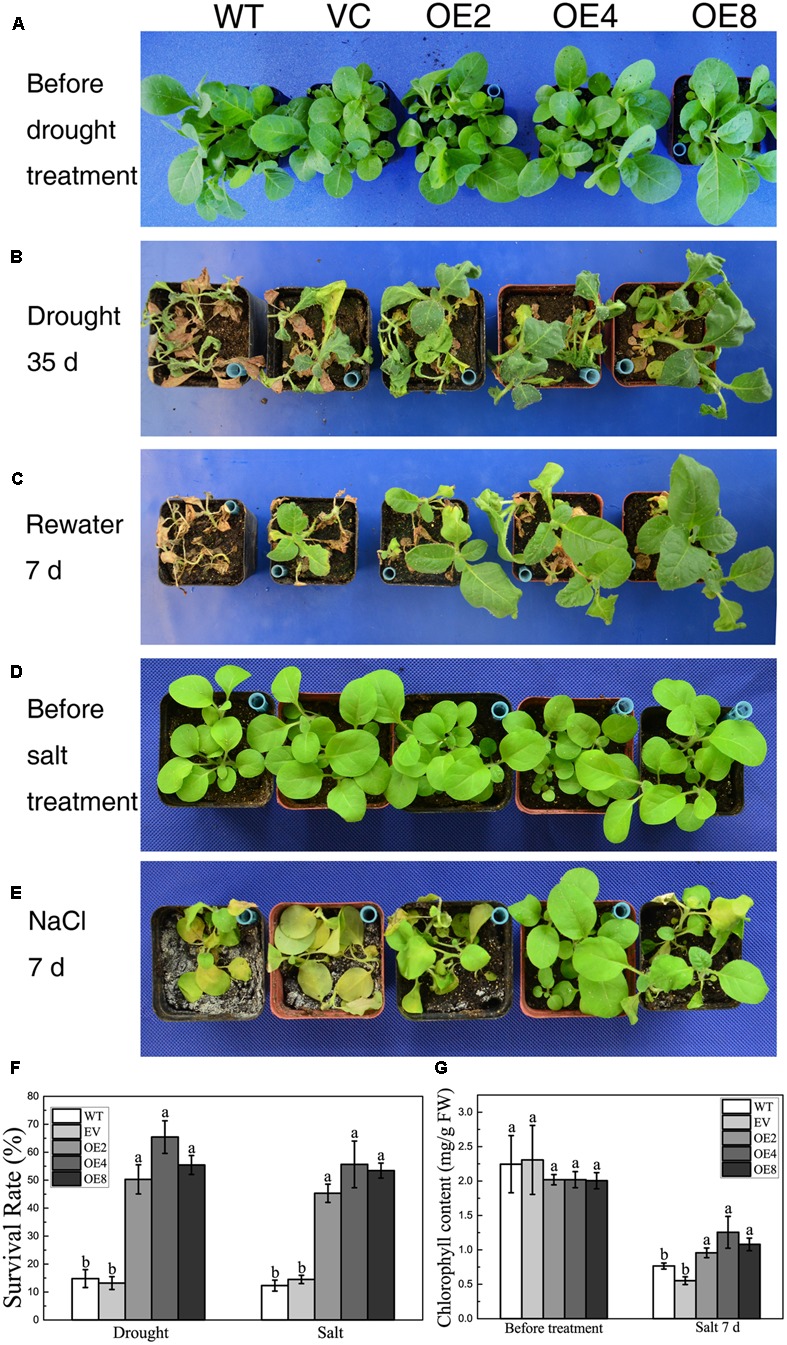
Analysis of the drought and salt tolerance in control plants and transgenic plants overexpressing *BdCIPK31*. **(A,D)** Four-week-old tobacco plants grown in normal condition. **(B)** Plants were subjected to drought for 35 days. **(C)** Plants in **(B)** were re-watered for 7 days. **(E)** Plants were subjected to high salinity for 7 days. **(F)** Statistical analysis of survival rates. **(G)** Chlorophyll content analysis of plants subjected to salt treatment. Data in **(F,G)** represent the means ± SE from three independent replicates. Different letters represent significant difference in each condition (Duncan’s test, *P* < 0.05).

BdCIPK31 also functioned in stress tolerance during vegetative growth. Drought stress was given to 4-week-old plants. Both controls and OE lines shriveled after a 35 days exposure to drought. However, the control lines were more severely wilted, whereas the leaves of the OE lines remained expanded (**Figure 4B**). After re-watering for 7 days, more than 50% of the OE plants survived compared with only 14.8% of the WT plants. Meanwhile, 13.2% of VC plants were recovered from drought (**Figures 4C,F**).

In salt treatment, most of the control plants displayed languish and chlorosis after a 7-day-exposure to salt, whereas the OE plants remained thriving and green (**Figures 4D,E**). Less than 15% of the control seedlings survived. By contrast, the survival rates of the OE lines were 45% (OE2), 55% (OE4), and 53% (OE8), respectively (**Figure 4F**). Moreover, chlorophyll content analysis showed that the OE plants contained more chlorophyll than the control after salt treatment (**Figure 4G**). These results suggest that BdCIPK31 improved drought and high-salinity tolerance in transgenic tobacco.

### Overexpression of *BdCIPK31* Confers Hypersensitivity to Exogenous ABA in Tobacco but Does Not Affect ABA Biosynthesis under Drought and Salt Stress

**FIGURE 5:**
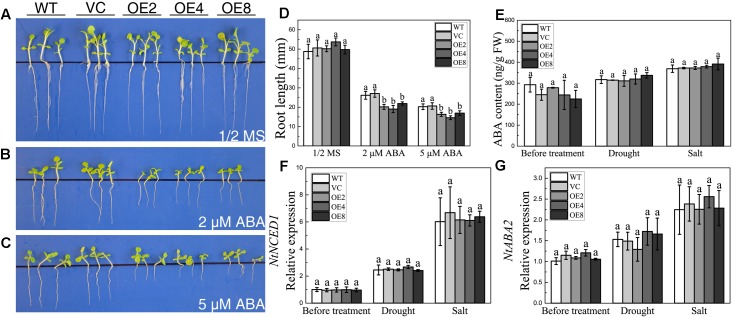
Analyses of the plant sensitivity exogenous ABA, ABA content, and ABA synthesis-related gene expression and under drought and salt treatments. Seedlings grown on **(A)** 1/2 MS medium, **(B,C)** 1/2 MS medium with **(B)** 2 μM or **(C)** 5 μM ABA for 21 days. **(D)** Root length analysis of control and OE seedlings. **(E)** Endogenous ABA level of control and OE plants before and after treatments. **(F,G)** Expression levels of **(F)** *NtNCED1* and **(G)** *NtABA2* in control and OE plants before and after treatments. Data in **(D–G)** represent the means ± SE from three independent replicates. Different letters represent significant difference in each condition (Duncan’s test, *P* < 0.05).

The downregulation of *BdCIPK31* in response to exogenous ABA implied that BdCIPK31 might participate in ABA signaling. Seedlings were grown on the 1/2 MS supplied with 2 or 5 μM ABA for 21 days. All seedlings exhibited growth inhibition under ABA treatment. However, the OE lines showed shorter root lengths than those of the control lines (**Figures 5A–D**). The result indicates that *BdCIPK31* overexpression increases plant sensitivity to ABA.

To investigate whether BdCIPK31 affected ABA biosynthesis, endogenous ABA levels were examined under drought or salt condition. Interestingly, the endogenous ABA contents in the OE seedlings were similar to those in the controls after treatment (**Figure 5E**). The key enzymes in ABA biosynthesis were also examined at the transcript level. The transcripts of *NtNCED1* and *NtABA2* in the OE seedlings were similar to those in the controls under both drought and salt stress (**Figures 5F,G**). These findings suggest that BdCIPK31 has no effect on ABA biosynthesis, and BdCIPK31 may function in the downstream of ABA.

### Overexpression of *BdCIPK31* Confers Better Water Status and Decreased Stomatal Aperture in Response to Drought Stress

**FIGURE 6:**
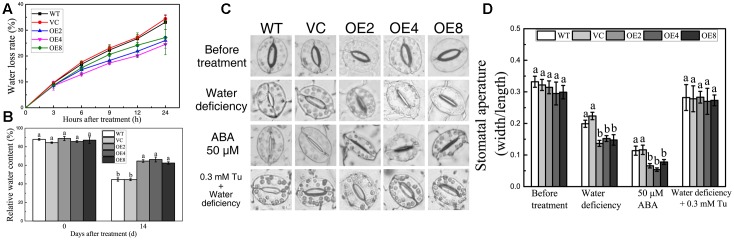
Analyses of RWC, water loss, and stomatal aperture. **(A)** Water loss rate of control and OE leaves in 24 h. **(B)** RWC of control and OE plants after drought treatment. Data in **(A,B)** represent the means ± SE from three independent replicates. **(C)** Stomatal closure under water deficiency, ABA, and Tu treatments. **(D)** Statistical analysis of the stomatal width:length ratio. Data in **(D)** represent the means ± SE from three independent replicates. Different letters represent significant difference in each condition (Duncan’s test, *P* < 0.05).

Enhanced drought tolerance implied the differences in water status between the OE and control plants. Fully expanded leaves were obtained in normal growth condition, and the FW changes of the leaves were measured regularly in 24 h. The water loss rates were calculated, and the OE lines displayed significantly lower water loss rates than the controls (**Figure 6A**). RWCs were also measured. After 14 days of drought treatment, the RWC of the control lines was 20% lower than that of controls (**Figure 6B**).

Transpiration through stomata is the main way of water loss under water-deficient conditions (Li et al., 2013). Thus, we investigated whether BdCIPK31 affects stomatal closure under drought stress. The stomatal width:length ratio was adopted as the index of stomatal closure. The leaves of all the lines were floated in buffer solution under light to cause the stomata to fully open prior to treatment application (**Figure 6C**). After exposing to low-humidity environment for 2 h, the OE lines displayed lower stomatal width:length ratios (**Figures 6C,D**).

Since stomatal closure is mediated by ABA, we performed an investigation on whether BdCIPK31 functioned in ABA-mediated stomatal closure. Leaves were incubated in 25 or 50 μM ABA solution for 4 h, and the OE lines displayed smaller stomatal aperture than the controls (**Figures 6C,D**). However, when the leaves were pretreated by Tu, the humidity-induced stomatal closure was inhibited, no significant differences in stomatal aperture between control and transgenic lines were observed (**Figures 6C,D**). These findings suggest that BdCIPK31 participates in ABA-mediated stomatal closure.

### Overexpression of *BdCIPK31* Affects Plant Ion Homeostasis under Salt Stress

**FIGURE 7:**
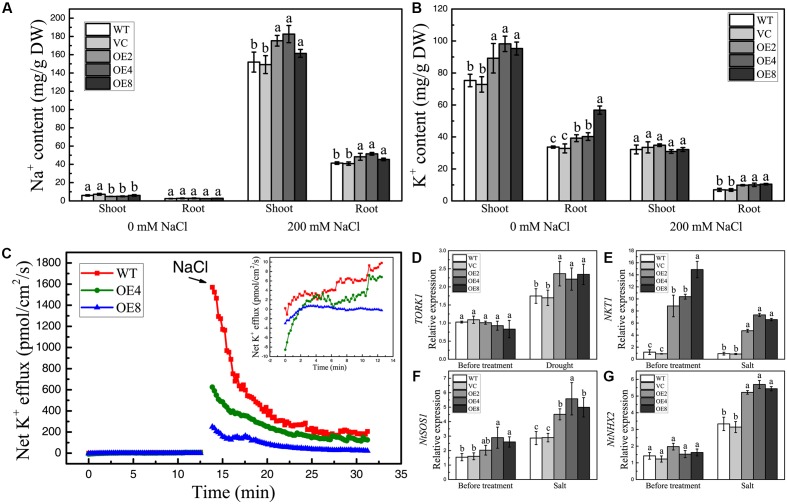
Analyses of ion content, K^+^ efflux in seedlings under salt condition, and expression analyses of ion-transporter genes in plants. **(A)** Na^+^ and **(B)** K^+^ levels in shoots and roots of control and OE seedlings under salt stress. **(C)** Net K^+^ efflux in root tips of WT and OE seedlings at the addition of 150 mM NaCl. The insert shows the K^+^ efflux before NaCl addition. **(D)** Expression levels of *TORK1* in the leaves of control and OE plants under drought treatment. **(E–G)** Expression levels of **(E)** *NKT1*, **(F)** *NtSOS1*, and **(G)** *NtNHX2* in the control and OE young seedlings under salt stress. Data in **(A,B,D–G)** represent the means ± SE from three independent replicates. Different letters represent significant difference in each condition (Duncan’s test, *P* < 0.05).

High salinity can disrupt intracellular ion homeostasis. To determine whether BdCIPK31 influences ion accumulation under high-salinity conditions, the Na^+^ and K^+^ contents in shoots and roots were assessed. The Na^+^ levels were similar in all seedlings without treatment (**Figure 7A**). By contrast, the K^+^ contents were higher in OE lines (**Figure 7B**). After salt treatment, Na^+^ content increased, whereas K^+^ content decreased, in all plants. However, the OE seedlings contained more Na^+^ than the control in both shoots and roots (**Figure 7A**). Notably, the K^+^ level in the shoots of the OE seedlings was equal to that in the controls after salt treatment, whereas the roots of the OE plants accumulated more K^+^ than the control did (**Figure 7B**). These findings indicate that BdCIPK31 is involved in intracellular Na^+^/K^+^ homeostasis under salt stress. Further analysis of the salt-shock-induced K^+^ efflux in OE plants was performed. Before treatment, the WT seedlings showed a slightly higher K^+^ efflux compared with OE seedlings. After addition of 150 mM NaCl, the OE seedlings displayed significantly lower K^+^ efflux than the WT (**Figure 7C**). This result supports the notion that BdCIPK31 functions in preventing K^+^ loss from root under salt stress.

### Overexpression of *BdCIPK31* Modulates the Expression of Ion-Transporter Genes under Drought and Salt Stress

Ion channels and transporters play important roles in stomatal movement and intracellular ion homeostasis. To clarify whether BdCIPK31 affects ion-transporter gene expression, we measured the transcripts of tobacco K^+^ influx channel genes *NKT1*, *NKT2*, *NTKC1*, K^+^ efflux channel *TORK1*, Na^+^/H^+^ antiporters *NtSOS1*, and Na^+^, K^+^/H^+^ antiporter *NtNHX2*.

The outward K^+^ channels play essential roles in stomatal closure. TORK1 is the homolog of *Arabidopsis* guard cell K^+^ efflux channel GORK in *N. tabacum* (Eisenach et al., 2014). The transcripts of *TORK1* in the leaves of OE lines showed at least a 1.5-fold increase compared with that in the controls under drought conditions (**Figure 7D**). This finding implies a stronger K^+^ efflux in the guard cells of OE plants.

Selective uptake of K^+^ and extrusion or compartmentalization of Na^+^ are main strategies for plants to cope with ion stress caused by high salinity. In *Arabidopsis*, K^+^-channel proteins AKT1 and AtKC1 functions in K^+^ uptake (Hirsch et al., 1998; Reintanz et al., 2002; Pilot et al., 2003), where AKT2 participates in long-distance K^+^ transport (Lacombe et al., 2000). The expression levels of their homologs *NKT1*, *NKT2,* and *NTKC1* in tobacco were selected and examined under salt treatment together with the *NtSOS1* and *NtNHX2*. Results showed that *NKT1* exhibited significantly higher transcript levels in transgenic seedlings than in controls under normal and high-salinity conditions (**Figure 7E**). The transcripts of *NKT2* and *NtKC1* in OE seedlings were equal to those in the controls before and after treatment (data not shown). Moreover, the OE lines showed higher *NtSOS1* and *NtNHX2* expression levels than the controls only under NaCl treatment (**Figures 7F,G**). These findings demonstrate that BdCIPK31 affects the expressions of several ion channel and transporter genes under high-salinity stress.

### Overexpression of *BdCIPK31* Enhances the Plant’s Scavenging Ability for Reactive Oxygen Species (ROS) and Maintains Plasma Membrane Stability under Drought and Salt Stress

Both drought and salt stress can cause oxidative and osmotic damages to plants. To explore whether BdCIPK31 functions in resistance to these damages, we detected the malondialdehyde (MDA) content, the IL, and the H_2_O_2_ content. The results showed that the IL and MDA levels were lower in OE seedlings (Supplementary Figures S7A,B), and the OE plants accumulated less H_2_O_2_ than the controls (Supplementary Figure S7C), suggesting that the OE seedlings suffered milder damage than the control under drought and salt stress.

Reactive oxygen species are essential signal-transduction molecules as well as toxic by-products of stress metabolism (Huang et al., 2012). Antioxidative enzymes, such as CAT, POD, SOD, and GST, and antioxidants, such as anthocyanin and ascorbic acid, function in preventing damage from over-produced ROS (Julkowska and Testerink, 2015).

The activities of CAT, POD, SOD, and GST were examined, and the results showed that the activities of all these antioxidative enzymes were higher in the OE seedlings than that in the controls (Supplementary Figure S8). Meanwhile, the OE plants accumulated more ascorbic acid and anthocyanin than the controls after treatment (Supplementary Figure S8). Moreover, the expression levels of *NtCAT1*, *NtPOX2*, *NtSOD*, *NtGST*, ascorbate peroxidase gene *NtAPX*, and flavonoid biosynthetic gene *NtDFR* were also higher in OE plants. By contrast, the ROS-producer genes *NtRbohD* and *NtRbohF* showed lower transcript levels in OE plants (Supplementary Figure S8).

Osmolyte accumulation is an important way to balance cell osmotic pressure. The proline and soluble sugar contents were examined. The OE seedlings accumulated more proline and soluble sugar under treatments (Supplementary Figure S9).

The results of the increased osmolyte accumulation in OE plants were also confirmed at transcriptional level. The expression levels of *NtP5CS1*, which encodes a rate-limiting enzyme of proline biosynthesis and a tobacco sucrose synthase gene *NtSUS1* were detected, together with polyamine biosynthesis genes *NtADC1* and *NtSAMDC1*. All these genes exhibited higher transcript levels in OE seedlings under drought or salt stress (Supplementary Figure S9). The above results contribute to explain the enhancement of the stress tolerance in the transgenic tobacco.

### Overexpression of *BdCIPK31* Enhances Plant Tolerance to Oxidative Stress

**FIGURE 8:**
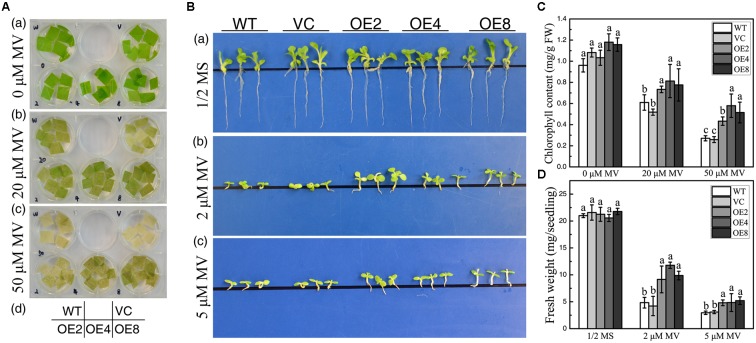
Analyses of oxidative tolerance control and OE plants. **(A)** Leaf disks obtained from control and OE seedlings treated with methyl viologen (MV). **(B)** Seedlings grown on 1/2 MS containing 0, 2, or 5 μM MV. **(C)** Chlorophyll content in leaves treated with 20 or 50 μM MV. **(D)** Average fresh weight of seedlings treated by 2 or 5 μM MV. Data in **(C,D)** represent the means ± SE from three independent replicates. Different letters represent significant difference in each condition (Duncan’s test, *P* < 0.05).

Overexpression of *BdCIPK31* confers enhanced ROS-scavenging ability in transgenic tobacco, which implies that BdCIPK31 may participate in plant response to oxidative stress. Leaf disks were incubated in 20 or 50 μM methyl viologen (MV) for 72 h. All leaf disks exhibited bleaching, and the controls displayed more severe results than those of the OE leaves (**Figure 8A**). Furthermore, when seedlings were grown on 1/2 MS media with 2 or 5 μM MV for 14 days, the OE lines disclosed a better phenotype than that of the controls (**Figure 8B**). These observed results were further confirmed by chlorophyll content and FW assay results (**Figures 8C,D**). All the results suggest that BdCIPK31 enhanced plant tolerance to oxidative stress.

### BdCIPK31 Modulates the Expressions of Some Stress-Related Genes in Transgenic Tobacco

**FIGURE 9:**
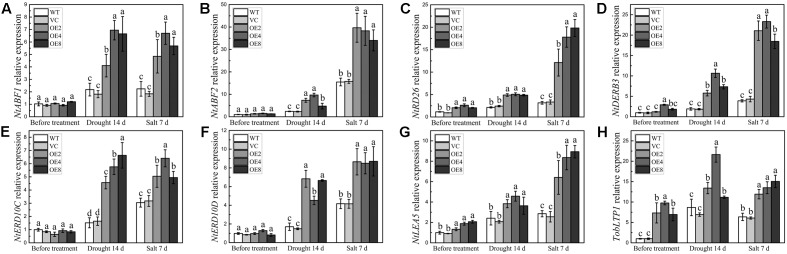
Expression analyses of stress-related genes in plants under drought and salt treatments. Expression levels of **(A)** *NtABF1*, **(B)** *NtABF2*, **(C)** *NtRD26*, **(D)** *NtDREB3*, **(E)** *NtERD10C*, **(F)** *NtERD10D*, **(G)** *NtLEA5*, and **(H)** *TobLTP1* were examined by qRT-PCR. Data represent the means ± SE from three independent replicates. Different letters represent significant difference in each condition (Duncan’s test, *P* < 0.05).

The transcripts of some stress-related genes in OE and control plants were also examined under drought or salt treatment. The selected genes included stress-responsive transcription factor genes, such as *NtABF1*, *NtABF2*, *NtDREB3*, and *NtRD26*, and stress-defense genes, such as *NtERD10C*, *NtERD10D*, *NtLEA5*, and *TobLTP1*. The transcripts of all these tested genes were higher in the OE plants under stress conditions (**Figures 9A–H**). Notably, the transcripts of *NtRD26*, *NtDREB3*, *NtLEA5,* and *TobLTP1* were evidently higher in the OE plants than in the controls before and after treatments (**Figures 9B–D,G,H**). These findings suggest that BdCIPK31 can elevate the expression levels of stress signal transduction related genes together with stress-defense genes under drought and salt conditions.

## Discussion

### BdCIPK31 Is a Positive Regulator in Plant Response to Drought, High Salinity, and ABA

Previous studies on *CIPKs* revealed that numerous *CIPK* genes regulate plant responses to abiotic stress. In our previous study, *TaCIPK14* and *TaCIPK29* were found to confer single or multiple stress tolerance in transgenic tobacco (Deng et al., 2013a,b). In the present work, overexpression of *BdCIPK31*, a stress-responsive *CIPK* gene, conferred improved drought and salt tolerance in transgenic tobacco (**Figures 3**, **4**). These findings demonstrate that BdCIPK31 has a positive function in plant responses to drought and high salinity.

Abscisic acid is a pivotal phytohormone in plant stress responses (Finkelstein et al., 2002), which plays a crucial role in the coordination of plant growth and stress tolerance (Raghavendra et al., 2010). It was previously demonstrated that CIPKs participate in stress response through the ABA signaling pathway (Chen et al., 2012; Wang et al., 2016; Zhou et al., 2016). In the present study, BdCIPK31 increased plant sensitivity to ABA (**Figure 5**), and accelerated ABA-mediated stomatal closure (**Figures 6C,D**). Moreover, when ABA biosynthesis inhibitor Tu was applied together with osmotic and salt treatments (Yan et al., 2014), the phenotype of enhanced tolerance in OE lines disappeared (**Figures 3G,H**). Furthermore, BdCIPK31 exerted no effect on ABA biosynthesis (**Figures 5E–G**), indicating that the alternation of ABA content is not the key reason for stress tolerance in *BdCIPK31*-overexpressing tobacco. The above results demonstrate that BdCIPK31 serves as a positive regulator in ABA signaling pathway. Moreover, the improved stress tolerance in transgenic tobacco is dependent on endogenous ABA.

Our results indicated that the BdCIPK31 was a positive regulator in stress and ABA response, while *BdCIPK31* was downregulated by abiotic stress and ABA, these were unexpected. However, similar discrepancies are not rare in studies of *CIPK* and other gene families. *OsCIPK31* and *OsCIPK03* were the homologs of *BdCIPK31* in rice. The stress-repressed *OsCIPK31* gene functioned positively in stress response, whereas the stress-induced *OsCIPK03* gene conferred stress sensitivity to transgenic rice (Piao et al., 2010; Rao et al., 2011). Similar contradictions were also reported in other gene families of different species. *ONAC095*, a NAC transcription factor gene in rice, was induced by drought and ABA, repressed by low temperature. However, overexpressing *ONAC095* in rice showed no effect on drought tolerance, while the dominant chimeric repressor-mediated suppression of *ONAC095* enhanced drought tolerance and ABA sensitivity (Huang et al., 2016).

### BdCIPK31 Improves Plant Tolerance to Water Stress

Under water-deficient conditions, plants need to maintain favorable water status. Transpiration from stomata is the major means to eliminate water (Li et al., 2013), and ABA-mediated stomatal movement is a crucial process in preventing water loss (Leung and Giraudat, 1998; Pitzschke and Hirt, 2009). ABA-mediated stomatal movement is associated with the K^+^ flux in guard cells (Hosy et al., 2003; Kim et al., 2010). CBL-CIPK networks were reported to function in the stomatal movements. AtCIPK23, together with AtCBL1 and AtCBL9, activates the K^+^ channel AKT1 and affects stomatal closure (Li et al., 2006; Xu et al., 2006; Cheong et al., 2007; Lee et al., 2007). In this study, overexpression of *BdCIPK31* improved the sensitivity of stomatal closure to drought treatment, and ABA is confirmed to be involved in this process (**Figures 6C,D**). This finding confirmed that BdCIPK31 could promote stomatal closure via the ABA signaling pathway. Thus, the OE seedlings displayed lower water loss rate and higher RWC than the control plants under drought treatment (**Figures 6A,B**). This result indicates that the OE plants possessed a better water status. Moreover, the expression level of the guard cell K^+^ efflux channel gene *TORK1* elevated more obviously in the leaves of the OE plants under drought treatment, and the outflow of K^+^ from guard cells can cause stomatal closing (Ache et al., 2000; Hosy et al., 2003; Osakabe et al., 2013). Hence, the above findings indicate that BdCIPK31 plays a positive role in stomatal closure.

### BdCIPK31 Improves Plant Tolerance to Ionic Stress

High salinity causes ionic stress to plants. High Na^+^ concentration in growth medium disrupts plant ion uptake and leads to an abnormal Na^+^ accumulation, whereas the K^+^ accumulation decreases (Bartels and Sunkar, 2005). Excess Na^+^ in the cytoplasm is toxic, whereas potassium is essential for many biochemical and physiological processes. The chief mechanisms maintaining intracellular K^+^/Na^+^ homeostasis include restricting Na^+^ uptake by selective ion uptake, and reducing cytoplasmic Na^+^ content by Na^+^ extrusion and/or intracellular Na^+^ compartmentalization (Lv et al., 2012). These mechanisms must be implemented by regulating ion channels and transporters. Earlier studies revealed that CBL-CIPK networks function in regulating ion transport under salt stress. The classical *Arabidopsis* SOS pathway functions in extruding Na^+^ (Liu and Zhu, 1997, 1998; Qiu et al., 2002). Transgenic tobacco overexpressing *TaCIPK14* and *TaCIPK29* can maintain higher K^+^/Na^+^ ratios under salt treatment (Deng et al., 2013a,b). Furthermore, the AtCIPK23 can activate the K^+^ influx channel AKT1 in the root, leading to an influx of K^+^ (Li et al., 2006; Cheong et al., 2007; Lee et al., 2007). Therefore, we tried to clarify whether BdCIPK31 functions in intracellular ion homeostasis under salt stress. Interestingly, transgenic plants overexpressing *BdCIPK31* retained significantly more Na^+^ than the control, despite the former’s salt-tolerant phenotype (**Figure 7A**). Similar phenomena have also been reported previously. The *atcbl10* mutant shows hypersensitivity to salt stress, but the Na^+^ content of this mutant is significantly lower than that in the control. Further investigations indicate that AtCIPK24 interacts with AtCBL10, and the complex is involved in intracellular Na^+^ compartmentalization (Kim et al., 2007). Transgenic tomatos overexpressing *SlCIPK24* display more tolerance to salt, and the enhanced tolerance is associated with higher Na^+^ level in aerial parts. *SlCIPK24* induces and upregulates SlSOS1, endosomal K^+^, Na^+^/H^+^ antiporter LeNHX2, and vacuolar Na^+^/H^+^ antiporter LeNHX4, indicating that SlCIPK24 functions in Na^+^ partitioning and compartmentalization under salt stress (Huertas et al., 2012). In our work, *BdCIPK31* overexpression upregulated the transcripts of *NtSOS1* and *NtNHX2* under salt stress (**Figures 7F,G**). This finding is consistent with the higher sodium content in *BdCIPK31*-overexpressing plants relative to the control, implying that BdCIPK31 may be involved in Na^+^ compartmentalization in endosomes or vacuoles under salt stress. Moreover, BdCIPK31 also functions in preventing K^+^ loss. Transgenic plants overexpressing *BdCIPK31* contained more K^+^ in whole seedlings under normal conditions (**Figure 7B**). Under salt treatment, although the OE seedlings retained more Na^+^ than the control (**Figure 7A**), OE seedlings still retained more K^+^ in roots, whereas the shoot K^+^ accumulation was similar between OE and control seedlings (**Figure 7B**). This result could be explained by the expression analyses of the K^+^ channel genes. Overexpression of *BdCIPK31* constitutively elevated the expression of *NKT1* (**Figure 7E**), and the NKT1 may function in root K^+^ uptake (Sano et al., 2007). However, BdCIPK31 does not affect the expression of *NKT2* that might be involved in long-distance K^+^ transport (Lacombe et al., 2000; Sano et al., 2007). Thus, the above results imply that BdCIPK31 might participate in K^+^ uptake in the root, but does not affect K^+^ transport from roots to shoots. Further investigation revealed that the OE plants exhibited lower net K^+^ efflux in roots than the WT (**Figure 7C**). The root plasma membrane is depolarized under salt treatment, thus the channel-mediated K^+^ uptake are inhibited in such condition (Shabala et al., 2016). Therefore, our finding implies that BdCIPK31 also functions in preventing K^+^ loss.

### BdCIPK31 Improves Plant Tolerance to Oxidative Stress

Exposure to drought or high salinity results in some common reactions in plants (Bartels and Sunkar, 2005), such as oxidative and osmotic stress. Both drought and salt stress leads to ROS overproduction, which acts as important stress signal-transduction molecules. However, excess ROS brings cell toxicity, leading to enzyme activity disruption, membrane injury, and cell death (Gouiaa et al., 2012). Thus, plants have developed an efficient antioxidative system, which includes ROS-scavenging enzymes and antioxidants, to regulate ROS content to appropriate levels (Ruiz-Lozano et al., 2012). CIPKs affect the antioxidant system in both physiologic and transcript levels have been reported previously (Yang et al., 2008; Piao et al., 2010; Chen et al., 2012; Deng et al., 2013a,b; Drerup et al., 2013; He et al., 2013). We demonstrated that overexpression of *BdCIPK31* significantly improved the ROS-scavenging system in both physiological and transcriptional level, whereas the transcripts of ROS-producer genes were downregulated (Supplementary Figure S8). As a result, the OE plants exhibited lower H_2_O_2_, MDA, and IL levels relative to the control (Supplementary Figure S7). Such finding indicates a decreased ROS accumulation and milder membrane damage in *BdCIPK31*-overexpressing plants. The above results indicate that BdCIPK31 functions in enhancing the plant ROS-scavenging system. Further study showed that *BdCIPK31* overexpression conferred enhanced tolerance to oxidative stress (**Figure 8**), confirming that BdCIPK31 participated in the antioxidation process.

### BdCIPK31 Improves Plant Tolerance to Osmotic Stress

Both drought and high salinity cause cell dehydration, which leads to osmotic stress (Bartels and Sunkar, 2005). Plants synthesize additional osmolytes to maintain turgor to cope with osmotic stress. In this study, the *BdCIPK31*-overexpressing tobacco exhibited higher proline and soluble sugar levels under stress together with higher transcripts of the *NtP5CS1* and *NtSUS1* genes (Supplementary Figures S9A–D). The transcripts of the genes on polyamine biosynthesis, *NtADC1* and *NtSAMDC1*, were also obviously elevated in the OE plants (Supplementary Figures S9E,F). These results indicate that *BdCIPK31* could enhance ROS-scavenging system to alleviate cell damage and enhance osmolyte synthesis to balance cellular osmotic pressure.

### BdCIPK31 Modulates the Expression of Stress-Related Genes

The expression of stress-related genes are modulated through ABA-dependent and ABA-independent pathways (Yamaguchi-Shinozaki and Shinozaki, 2006). The ABFs are the major transcription factors in the ABA-dependent pathway, whereas DREBs have key functions in ABA-independent pathway (Yoshida et al., 2014). In this study, the transcripts of *NtABF1*, *NtABF2*, and *NtRD26*, which encodes a NAC transcription factor in the ABA-dependent pathway (Fujita et al., 2004), were significantly higher in the *BdCIPK31*-overexpressing tobacco under drought and salt stress (**Figures 9A–C**). Moreover, *NtDREB3* also presented higher transcripts in OE seedlings under the same conditions (**Figure 9D**). The transcripts of the stress-inducible genes, including *NtERD10C*, *NtERD10D*, *NtLEA5*, and *TobLTP1*, were higher in the *BdCIPK31*-overexpressing plants under stress (**Figures 9E–H**). The *NtERD10C* and *NtERD10D* genes encode group 2 LEA proteins, and the *NtLEA5* gene encodes a group 5 LEA protein. Both *NtERD10C/D* and *NtLEA5* function in binding water, stabilizing enzyme and macromolecular structures, and then preventing cells from damages caused by abiotic stress (Becker et al., 1996; Hundertmark and Hincha, 2008; Liu et al., 2009). *TobLTP1* encodes a lipid-transfer protein, which participates in abiotic stress responses (Trevino and O’Connell, 1998; Zhang et al., 2005). The stress-inducible genes are recognized as targets of *ABF* and *DREB* genes (Shinozaki et al., 2003; Fujita et al., 2005); thus, these findings demonstrated that BdCIPK31 modulates the transcripts of stress-responsive genes through both ABA-dependent and ABA-independent pathways.

## Conclusion

In conclusion, BdCIPK31 confers enhanced drought and salt stress tolerance and ABA hypersensitivity in plants. Overexpressing *BdCIPK31* functions in stomatal closure, ion homeostasis, ROS scavenging, osmolyte biosynthesis, and transcriptional regulation of stress-related genes. Overall, BdCIPK31 functions in plant responses to drought and salt stress as a positive regulator in ABA-mediated Ca^2+^ signaling pathway.

## Author Contributions

QL performed all the experiments, analyzed the data and wrote the manuscript. QW generated the transgenic tobacco plants and helped to perform other experiments. RW, YZ, FZ, YH, SZ, and JF helped to perform the physiological experiments and promoter analysis. GH and GY conceived the study, designed experiments and wrote the manuscript.

## Conflict of Interest Statement

The authors declare that the research was conducted in the absence of any commercial or financial relationships that could be construed as a potential conflict of interest.

## Footnotes

1 http://blast.ncbi.nlm.nih.gov/Blast.cgi

2 http://plants.ensembl.org/index.html

3 http://bioinformatics.psb.ugent.be/webtools/plantcare/html/

## Abbreviations

ABA: abscisic acid
ABF: ABRE-binding factor
ABRE: ABA-responsive element
CaMV: *Cauliflower mosaic virus*
CAT: catalase
CBL: calcineurin B-like protein
CDPK: Ca-dependent protein kinase
CIPK: CBL-interacting protein kinase
DREB: DRE-/CRT-binding protein
GST: glutathione *S*-transferase
IL: ion leakage
MDA: malondialdehyde
MS: Murashige and Skoog
POD: peroxidase
qRT-PCR: quantitative reverse-transcription PCR
ROS: reactive oxygen species
RWC: relative water content
SOD: superoxide dismutase
SOS: salt overly sensitive
Tu: tungstate sodium
VC: vector control
WT: wild type.

## References

[B1] Ache P. Becker D. Ivashikina N. Dietrich P. Roelfsema M. R. G. Hedrich R. (2000). GORK, a delayed outward rectifier expressed in guard cells of *Arabidopsis thaliana*, is a K^+^-selective, K^+^-sensing ion channel. *FEBS Lett.* 486 93–98. 10.1016/s0014-5793(00)02248-1 11113445

[B2] Asano T. Tanaka N. Yang G. Hayashi N. Komatsu S. (2005). Genome-wide identification of the rice calcium-dependent protein kinase and its closely related kinase gene families: comprehensive analysis of the CDPKs gene family in rice. *Plant Cell Physiol.* 46 356–366. 10.1093/pcp/pci035 15695435

[B3] Bartels D. Sunkar R. (2005). Drought and salt tolerance in plants. *Crit. Rev. Plant Sci.* 24 23–58. 10.1080/07352680590910410

[B4] Batistic O. Kudla J. (2004). Integration and channeling of calcium signaling through the CBL calcium sensor/CIPK protein kinase network. *Planta* 219 915–924. 10.1007/s00425-004-1333-3 15322881

[B5] Becker W. Heukelbach J. Kentrup H. Joost H. G. (1996). Molecular cloning and characterization of a novel mammalian protein kinase harboring a homology domain that defines a subfamily of serine/threonine kinases. *Eur. J. Biochem.* 235 736–743. 8654423 10.1111/j.1432-1033.1996.00736.x

[B6] Chen L. Ren F. Zhou L. Wang Q. Q. Zhong H. Li X. B. (2012). The *Brassica napus* calcineurin B-Like 1/CBL-interacting protein kinase 6 (CBL1/CIPK6) component is involved in the plant response to abiotic stress and ABA signalling. *J. Exp. Bot.* 63 6211–6222. 10.1093/jxb/ers273 23105131 PMC3481211

[B7] Chen X. Gu Z. Xin D. Hao L. Liu C. Huang J. (2011). Identification and characterization of putative CIPK genes in maize. *J. Genet. Genomics* 38 77–87. 10.1016/j.jcg.2011.01.005 21356527

[B8] Cheong Y. H. Pandey G. K. Grant J. J. Batistic O. Li L. Kim B.-G. (2007). Two calcineurin B-like calcium sensors, interacting with protein kinase CIPK23, regulate leaf transpiration and root potassium uptake in Arabidopsis. *Plant J.* 52 223–239. 10.1111/j.1365-313X.2007.03236.x 17922773

[B9] Cui L.-G. Shan J.-X. Shi M. Gao J.-P. Lin H.-X. (2014). The miR156-SPL9-DFR pathway coordinates the relationship between development and abiotic stress tolerance in plants. *Plant J.* 80 1108–1117. 10.1111/tpj.12712 25345491

[B10] D’Angelo C. Weinl S. Batistic O. Pandey G. K. Cheong Y. H. Schultke S. (2006). Alternative complex formation of the Ca^2+^-regulated protein kinase CIPK1 controls abscisic acid-dependent and independent stress responses in Arabidopsis. *Plant J.* 48 857–872. 10.1111/j.1365-313X.2006.02921.x 17092313

[B11] Deng X. Hu W. Wei S. Zhou S. Zhang F. Han J. (2013a). TaCIPK29, a CBL-interacting protein kinase gene from wheat, confers salt stress tolerance in transgenic tobacco. *PLoS ONE* 8:e69881 10.1371/journal.pone.0069881 PMC3726728 23922838

[B12] Deng X. Zhou S. Hu W. Feng J. Zhang F. Chen L. (2013b). Ectopic expression of wheat TaCIPK14, encoding a calcineurin B-like protein-interacting protein kinase, confers salinity and cold tolerance in tobacco. *Physiol. Plant.* 149 367–377. 10.1111/ppl.12046 23534344

[B13] Drerup M. M. Schlucking K. Hashimoto K. Manishankar P. Steinhorst L. Kuchitsu K. (2013). The Calcineurin B-like calcium sensors CBL1 and CBL9 together with their interacting protein kinase CIPK26 regulate the *Arabidopsis* NADPH oxidase RBOHF. *Mol. Plant* 6 559–569. 10.1093/mp/sst009 23335733

[B14] Eisenach C. Papanatsiou M. Hillert E. K. Blatt M. R. (2014). Clustering of the K^+^ channel GORK of Arabidopsis parallels its gating by extracellular K^+^. *Plant J.* 78 203–214. 10.1111/tpj.12471 24517091 PMC4309415

[B15] Finkelstein R. R. Gampala S. S. L. Rock C. D. (2002). Abscisic acid signaling in seeds and seedlings. *Plant Cell* 14 S15–S45. 10.1105/tpc.010441 12045268 PMC151246

[B16] Fujita M. Fujita Y. Maruyama K. Seki M. Hiratsu K. Ohme-Takagi M. (2004). A dehydration-induced NAC protein, RD26, is involved in a novel ABA-dependent stress-signaling pathway. *Plant J.* 39 863–876. 10.1111/j.1365-313X.2004.02171.x 15341629

[B17] Fujita Y. Fujita M. Satoh R. Maruyama K. Parvez M. M. Seki M. (2005). AREB1 is a transcription activator of novel ABRE-dependent ABA signaling that enhances drought stress tolerance in *Arabidopsis*. *Plant Cell* 17 3470–3488. 10.1105/tpc.105.035659 16284313 PMC1315382

[B18] Gouiaa S. Khoudi H. Leidi E. O. Pardo J. M. Masmoudi K. (2012). Expression of wheat Na^+^/H^+^ antiporter *TNHXS1* and H^+^- pyrophosphatase *TVP1* genes in tobacco from a bicistronic transcriptional unit improves salt tolerance. *Plant Mol. Biol.* 79 137–155. 10.1007/s11103-012-9901-6 22415161

[B19] He L. Yang X. Wang L. Zhu L. Zhou T. Deng J. (2013). Molecular cloning and functional characterization of a novel cotton CBL-interacting protein kinase gene (GhCIPK6) reveals its involvement in multiple abiotic stress tolerance in transgenic plants. *Biochem. Biophys. Res. Commun.* 435 209–215. 10.1016/j.bbrc.2013.04.080 23660187

[B20] Heath R. L. Packer L. (1968). Photoperoxidation in isolated chloroplasts. I. Kinetics and stoichiometry of fatty acid peroxidation. *Arch. Biochem. Biophys.* 125 189–198. 10.1016/0003-9861(68)90654-1 5655425

[B21] Held K. Pascaud F. Eckert C. Gajdanowicz P. Hashimoto K. Corratge-Faillie C. (2011). Calcium-dependent modulation and plasma membrane targeting of the AKT2 potassium channel by the CBL4/CIPK6 calcium sensor/protein kinase complex. *Cell Res.* 21 1116–1130. 10.1038/cr.2011.50 21445098 PMC3193494

[B22] Hirsch R. E. Lewis B. D. Spalding E. P. Sussman M. R. (1998). A role for the AKT1 potassium channel in plant nutrition. *Science* 280 918–921. 10.1126/science.280.5365.918 9572739

[B23] Horsch R. B. Fry J. E. Hoffmann N. L. Eichholtz D. Rogers S. C. Fraley R. T. (1985). A simple and general method for transferring genes into plants. *Science* 227 1229–1231. 17757866 10.1126/science.227.4691.1229

[B24] Hosy E. Vavasseur A. Mouline K. Dreyer I. Gaymard F. Poree F. (2003). The *Arabidopsis* outward K^+^ channel *GORK* is involved in regulation of stomatal movements and plant transpiration. *Proc. Natl. Acad. Sci. U.S.A.* 100 5549–5554. 10.1073/pnas.0733970100 12671068 PMC154382

[B25] Hu W. Huang C. Deng X. Zhou S. Chen L. Li Y. (2013). TaASR1, a transcription factor gene in wheat, confers drought stress tolerance in transgenic tobacco. *Plant Cell Environ.* 36 1449–1464. 10.1111/pce.12074 23356734

[B26] Hu Y. Chen L. G. Wang H. P. Zhang L. P. Wang F. Yu D. Q. (2013). Arabidopsis transcription factor WRKY8 functions antagonistically with its interacting partner VQ9 to modulate salinity stress tolerance. *Plant J.* 74 730–745. 10.1111/tpj.12159 23451802

[B27] Huang G. T. Ma S. L. Bai L. P. Zhang L. Ma H. Jia P. (2012). Signal transduction during cold, salt, and drought stresses in plants. *Mol. Biol. Rep.* 39 969–987. 10.1007/s11033-011-0823-1 21573796

[B28] Huang L. Hong Y. Zhang H. Li D. Song F. (2016). Rice NAC transcription factor ONAC095 plays opposite roles in drought and cold stress tolerance. *BMC Plant Biol.* 16:203 10.1186/s12870-016-0897-y PMC5029094 27646344

[B29] Huertas R. Olias R. Eljakaoui Z. Galvez F. J. Li J. De Morales P. A. (2012). Overexpression of SlSOS2 (SlCIPK24) confers salt tolerance to transgenic tomato. *Plant Cell Environ.* 35 1467–1482. 10.1111/j.1365-3040.2012.02504.x 22390672

[B30] Hundertmark M. Hincha D. K. (2008). LEA (late embryogenesis abundant) proteins and their encoding genes in *Arabidopsis thaliana*. *BMC Genomics* 9:118 10.1186/1471-2164-9-118 PMC2292704 18318901

[B31] Jefferson R. A. Kavanagh T. A. Bevan M. W. (1987). GUS fusions: beta-glucuronidase as a sensitive and versatile gene fusion marker in higher plants. *EMBO J.* 6 3901–3907. 3327686 10.1002/j.1460-2075.1987.tb02730.x PMC553867

[B32] Jin X. Sun T. Wang X. T. Su P. P. Ma J. F. He G. Y. (2016). Wheat CBL-interacting protein kinase 25 negatively regulates salt tolerance in transgenic wheat. *Sci. Rep.* 6:28884 10.1038/srep28884 PMC4928124 27358166

[B33] Julkowska M. M. Testerink C. (2015). Tuning plant signaling and growth to survive salt. *Trends Plant Sci.* 20 586–594. 10.1016/j.tplants.2015.06.008 26205171

[B34] Kim B. G. Waadt R. Cheong Y. H. Pandey G. K. Dominguez-Solis J. R. Schultke S. (2007). The calcium sensor CBL10 mediates salt tolerance by regulating ion homeostasis in Arabidopsis. *Plant J.* 52 473–484. 10.1111/j.1365-313X.2007.03249.x 17825054

[B35] Kim K. N. Cheong Y. H. Grant J. J. Pandey G. K. Luan S. (2003). *CIPK3*, a calcium sensor-associated protein kinase that regulates abscisic acid and cold signal transduction in Arabidopsis. *Plant Cell* 15 411–423. 10.1105/tpc.006858 12566581 PMC141210

[B36] Kim T. H. Boehmer M. Hu H. Nishimura N. Schroeder J. I. (2010). Guard cell signal transduction network: advances in understanding abscisic acid, CO_2_, and Ca^2+^ signaling. *Annu. Rev. Plant Biol.* 61 561–591. 10.1146/annurev-arplant-042809-112226 20192751 PMC3056615

[B37] Kolukisaoglu U. Weinl S. Blazevic D. Batistic O. Kudla J. (2004). Calcium sensors and their interacting protein kinases: genomics of the Arabidopsis and rice CBL-CIPK signaling networks. *Plant Physiol.* 134 43–58. 10.1104/pp.103.033068 14730064 PMC316286

[B38] Kong X. Pan J. Zhang M. Xing X. Zhou Y. Liu Y. (2011). *ZmMKK4*, a novel group C mitogen-activated protein kinase kinase in maize (*Zea mays*), confers salt and cold tolerance in transgenic *Arabidopsis*. *Plant Cell Environ.* 34 1291–1303. 10.1111/j.1365-3040.2011.02329.x 21477122

[B39] Kudla J. Xu Q. Harter K. Gruissem W. Luan S. (1999). Genes for calcineurin B-like proteins in *Arabidopsis* are differentially regulated by stress signals. *Proc. Natl. Acad. Sci. U.S.A.* 96 4718–4723. 10.1073/pnas.96.8.4718 10200328 PMC16398

[B40] Lacombe B. Pilot G. Michard E. Gaymard F. Sentenac H. Thibaud J. B. (2000). A shaker-like K^+^ channel with weak rectification is expressed in both source and sink phloem tissues of Arabidopsis. *Plant Cell* 12 837–851. 10.1105/tpc.12.6.837 10852932 PMC149088

[B41] Lee S. C. Lan W.-Z. Kim B.-G. Li L. Cheong Y. H. Pandey G. K. (2007). A protein phosphorylation/dephosphorylation network regulates a plant potassium channel. *Proc. Natl. Acad. Sci. U.S.A.* 104 15959–15964. 10.1073/pnas.0707912104 17898163 PMC2000415

[B42] Leung J. Giraudat J. (1998). Abscisic acid signal transduction. *Annu. Rev. Plant Physiol. Plant Mol. Biol.* 49 199–222. 10.1146/annurev.arplant.49.1.199 15012233

[B43] Li J. Besseau S. Toronen P. Sipari N. Kollist H. Holm L. (2013). Defense-related transcription factors WRKY70 and WRKY54 modulate osmotic stress tolerance by regulating stomatal aperture in *Arabidopsis*. *New Phytol.* 200 457–472. 10.1111/nph.12378 23815736 PMC4284015

[B44] Li L. Kim B.-G. Cheong Y. H. Pandey G. K. Luan S. (2006). A Ca^2+^ signaling pathway regulates a K^+^ channel for low-K response in *Arabidopsis*. *Proc. Natl. Acad. Sci. U.S.A.* 103 12625–12630. 10.1073/pnas.0605129103 16895985 PMC1567929

[B45] Liu J. Zhu J. K. (1997). An *Arabidopsis* mutant that requires increased calcium for potassium nutrition and salt tolerance. *Proc. Natl. Acad. Sci. U.S.A.* 94 14960–14964. 10.1073/pnas.94.26.14960 9405721 PMC25145

[B46] Liu J. Zhu J. K. (1998). A calcium sensor homolog required for plant salt tolerance. *Science* 280 1943–1945. 10.1126/science.280.5371.1943 9632394

[B47] Liu X. Wang Z. Wang L. Wu R. Phillips J. Deng X. (2009). LEA 4 group genes from the resurrection plant *Boea hygrometrica* confer dehydration tolerance in transgenic tobacco. *Plant Sci.* 176 90–98. 10.1016/j.plantsci.2008.09.012

[B48] Luan S. (2009). The CBL-CIPK network in plant calcium signaling. *Trends Plant Sci.* 14 37–42. 10.1016/j.tplants.2008.10.005 19054707

[B49] Lv S. Jiang P. Chen X. Fan P. Wang X. Li Y. (2012). Multiple compartmentalization of sodium conferred salt tolerance in *Salicornia europaea*. *Plant Physiol. Biochem.* 51 47–52. 10.1016/j.plaphy.2011.10.015 22153239

[B50] Osakabe Y. Arinaga N. Umezawa T. Katsura S. Nagamachi K. Tanaka H. (2013). Osmotic stress responses and plant growth controlled by potassium transporters in *Arabidopsis*. *Plant Cell* 25 609–624. 10.1105/tpc.112.105700 23396830 PMC3608781

[B51] Pandey G. K. Kanwar P. Singh A. Steinhorst L. Pandey A. Yadav A. K. (2015). Calcineurin B-like protein-interacting protein kinase CIPK21 regulates osmotic and salt stress responses in Arabidopsis. *Plant Physiol.* 169 780–792. 10.1104/pp.15.00623 26198257 PMC4577403

[B52] Piao H. L. Xuan Y. H. Park S. H. Je B. I. Park S. J. Park S. H. (2010). OsCIPK31, a CBL-interacting protein kinase is involved in germination and seedling growth under abiotic stress conditions in rice plants. *Mol. Cells* 30 19–27. 10.1007/s10059-010-0084-1 20652492

[B53] Pilot G. Gaymard F. Mouline K. Cherel I. Sentenac H. (2003). Regulated expression of *Arabidopsis* Shaker K^+^ channel genes involved in K^+^ uptake and distribution in the plant. *Plant Mol. Biol.* 51 773–787. 10.1023/a:1022597102282 12678562

[B54] Pitzschke A. Hirt H. (2009). Disentangling the complexity of mitogen-activated protein kinases and reactive oxygen species signaling. *Plant Physiol.* 149 606–615. 10.1104/pp.108.131557 19201916 PMC2633849

[B55] Qiu Q. S. Guo Y. Dietrich M. A. Schumaker K. S. Zhu J. K. (2002). Regulation of SOS1, a plasma membrane Na^+^/H^+^ exchanger in *Arabidopsis thaliana*, by SOS2 and SOS3. *Proc. Natl. Acad. Sci. U.S.A.* 99 8436–8441. 10.1073/pnas.122224699 12034882 PMC123085

[B56] Raghavendra A. S. Gonugunta V. K. Christmann A. Grill E. (2010). ABA perception and signalling. *Trends Plant Sci.* 15 395–401. 10.1016/j.tplants.2010.04.006 20493758

[B57] Rao X.-L. Zhang X.-H. Li R.-J. Shi H.-T. Lu Y.-T. (2011). A calcium sensor-interacting protein kinase negatively regulates salt stress tolerance in rice (*Oryza sativa*). *Funct. Plant Biol.* 38 441–450. 10.1071/fp10205 32480899

[B58] Reintanz B. Szyroki A. Ivashikina N. Ache P. Godde M. Becker D. (2002). AtKC1, a silent *Arabidopsis* potassium channel alpha-subunit modulates root hair K^+^ influx. *Proc. Natl. Acad. Sci. U.S.A.* 99 4079–4084. 10.1073/pnas.052677799 11904452 PMC122651

[B59] Ruiz-Lozano J. M. Porcel R. Azcon C. Aroca R. (2012). Regulation by arbuscular mycorrhizae of the integrated physiological response to salinity in plants: new challenges in physiological and molecular studies. *J. Exp. Bot.* 63 4033–4044. 10.1093/jxb/ers126 22553287

[B60] Sano T. Becker D. Ivashikina N. Wegner L. H. Zimmermann U. Roelfsema M. R. (2007). Plant cells must pass a K^+^ threshold to re-enter the cell cycle. *Plant J.* 50 401–413. 10.1111/j.1365-313X.2007.03071.x 17425714

[B61] Shabala S. Bose J. Fuglsang A. T. Pottosin I. (2016). On a quest for stress tolerance genes: membrane transporters in sensing and adapting to hostile soils. *J. Exp. Bot.* 67 1015–1031. 10.1093/jxb/erv465 26507891

[B62] Shinozaki K. Yamaguchi-Shinozaki K. Seki M. (2003). Regulatory network of gene expression in the drought and cold stress responses. *Curr. Opin. Plant Biol.* 6 410–417. 10.1016/S1369-5266(03)00092-X 12972040

[B63] Sparkes I. A. Runions J. Kearns A. Hawes C. (2006). Rapid, transient expression of fluorescent fusion proteins in tobacco plants and generation of stably transformed plants. *Nat. Protoc.* 1 2019–2025. 10.1038/nprot.2006.286 17487191

[B64] Trevino M. B. O’Connell M. A. (1998). Three drought-responsive members of the nonspecific lipid-transfer protein gene family in *Lycopersicon pennellii* show different developmental patterns of expression. *Plant Physiol.* 116 1461–1468. 10.1104/pp.116.4.1461 9536064 PMC35054

[B65] Tripathi V. Parasuraman B. Laxmi A. Chattopadhyay D. (2009). CIPK6, a CBL-interacting protein kinase is required for development and salt tolerance in plants. *Plant J.* 58 778–790. 10.1111/j.1365-313X.2009.03812.x 19187042

[B66] Vogel J. P. Garvin D. F. Mockler T. C. Schmutz J. Rokhsar D. Bevan M. W. (2010). Genome sequencing and analysis of the model grass *Brachypodium distachyon*. *Nature* 463 763–768. 10.1038/nature08747 20148030

[B67] Wang L. Hu W. Sun J. Liang X. Yang X. Wei S. (2015). Genome-wide analysis of SnRK gene family in *Brachypodium distachyon* and functional characterization of BdSnRK2.9. *Plant Sci.* 237 33–45. 10.1016/j.plantsci.2015.05.008 26089150

[B68] Wang R. K. Li L. L. Cao Z. H. Zhao Q. Li M. Zhang L. Y. (2012). Molecular cloning and functional characterization of a novel apple MdCIPK6L gene reveals its involvement in multiple abiotic stress tolerance in transgenic plants. *Plant Mol. Biol.* 79 123–135. 10.1007/s11103-012-9899-9 22382993

[B69] Wang Y. Sun T. Li T. T. Wang M. Yang G. X. He G. Y. (2016). A CBL-interacting protein kinase TaCIPK2 confers drought tolerance in transgenic tobacco plants through regulating the stomatal movement. *PLoS ONE* 11:e0167962 10.1371/journal.pone.0167962 PMC5148042 27936160

[B70] Xiang Y. Huang Y. Xiong L. (2007). Characterization of stress-responsive CIPK genes in rice for stress tolerance improvement. *Plant Physiol.* 144 1416–1428. 10.1104/pp.107.101295 17535819 PMC1914128

[B71] Xu J. Li H.-D. Chen L.-Q. Wang Y. Liu L.-L. He L. (2006). A protein kinase, interacting with two calcineurin B-like proteins, regulates K^+^ transporter AKT1 in *Arabidopsis*. *Cell* 125 1347–1360. 10.1016/j.cell.2006.06.011 16814720

[B72] Yamaguchi-Shinozaki K. Shinozaki K. (2006). Transcriptional regulatory networks in cellular responses and tolerance to dehydration and cold stresses. *Annu. Rev. Plant Biol.* 57 781–803. 16669782 10.1146/annurev.arplant.57.032905.105444

[B73] Yan H. Jia H. Chen X. Hao L. An H. Guo X. (2014). The cotton WRKY transcription factor GhWRKY17 functions in drought and salt stress in transgenic *Nicotiana benthamiana* through ABA signaling and the modulation of reactive oxygen species production. *Plant Cell Physiol.* 55 2060–2076. 10.1093/pcp/pcu133 25261532

[B74] Yang Q. Chen Z.-Z. Zhou X.-F. Yin H.-B. Li X. Xin X.-F. (2009). Overexpression of SOS (*Salt Overly Sensitive*) genes increases salt tolerance in transgenic *Arabidopsis*. *Mol. Plant* 2 22–31. 10.1093/mp/ssn058 19529826 PMC2639737

[B75] Yang W. Kong Z. Omo-Ikerodah E. Xu W. Li Q. Xue Y. (2008). Calcineurin B-like interacting protein kinase OsCIPK23 functions in pollination and drought stress responses in rice (*Oryza sativa* L.). *J. Genet. Genomics* 35 531–543, S1–S2. 10.1016/S1673-8527(08)60073-9 18804072

[B76] Yoshida T. Mogami J. Yamaguchi-Shinozaki K. (2014). ABA-dependent and ABA-independent signaling in response to osmotic stress in plants. *Curr. Opin. Plant Biol.* 21 133–139. 10.1016/j.pbi.2014.07.009 25104049

[B77] Yu Q. An L. Li W. (2014). The CBL-CIPK network mediates different signaling pathways in plants. *Plant Cell Rep.* 33 203–214. 10.1007/s00299-013-1507-1 24097244

[B78] Zhang H. Yang B. Liu W. Z. Li H. Wang L. Wang B. (2014). Identification and characterization of CBL and CIPK gene families in canola (*Brassica napus* L.). *BMC Plant Biol.* 14:8 10.1186/1471-2229-14-8. PMC3890537 24397480

[B79] Zhang X. Zhang Z. J. Chen J. Chen Q. Wang X. C. Huang R. F. (2005). Expressing TERF1 in tobacco enhances drought tolerance and abscisic acid sensitivity during seedling development. *Planta* 222 494–501. 10.1007/s00425-005-1564-y 15871029

[B80] Zhou L. Lan W. Chen B. Fang W. Luan S. (2015). A calcium sensor-regulated protein kinase, CALCINEURIN B-LIKE PROTEIN-INTERACTING PROTEIN KINASE19, is required for pollen tube growth and polarity. *Plant Physiol.* 167 1351–1360. 10.1104/pp.114.256065 25713341 PMC4378171

[B81] Zhou Y. L. Sun X. D. Yang Y. Q. Li X. Cheng Y. Yang Y. P. (2016). Expression of *Stipa purpurea* *SpCIPK26* in *Arabidopsis thaliana* enhances salt and drought tolerance and regulates abscisic acid signaling. *Int. J. Mol. Sci.* 17:966 10.3390/Ijms17060966 PMC4926498 27338368

