# Supplementary material for: BdCIPK31, a Calcineurin B-Like Protein-Interacting Protein Kinase, Regulates Plant Response to Drought and Salt Stress
Source: Front Plant Sci. 2017 Jul 7;8:1184. doi: 10.3389/fpls.2017.01184 (PMC5500663; doi:10.3389/fpls.2017.01184)
Supplement: Supplementary file 1 [file Table_1.PDF]

Table S1. Primers used in gene amplification and plasmid construction

Primer	Sequence (5'-3')	Purpose
P1	F:GCAGATCTGATGTACAAGGCTAAGAGGGC<br>R: GCACTAGTTGCTGTGGAGCTTTTTTCTT	Introducing *BdCIPK31* coding sequence to pCambia 1303
P2	F:CCCGCTCTGACCAGTCATAAA<br>R: AAGCTGCCCTCTTAGCCTTG	*BdCIPK31* promoter amplification
P3	F: CCCAAGCTTTAGCCACACAAATGAGTT<br>R: CGGGATCCCTTCCAAGATAGGAGTAG	Introducing *BdCIPK31* promoter to pBI121
BdCIPK31-AD	F: CATATGATGTACAAGGCTAAGAGG<br>R: CTCGAGTGCTGTGGAGCTTTTTTC	Introducing *BdCIPK31* to pGADT7
BdCBL1-BD	F: CATATGATGGGGTGCTTCCATTCC<br>R: GAATTCTGTCACGATATCATCAAC	Introducing *BdCBL1* to pGBKT7
BdCBL2-BD	F: GAATTCATGGGGTGCATCCAGTCG<br>R: GGATCCTGTGACGATATCATCGAC	Introducing *BdCBL2* to pGBKT7
BdCBL3-BD	F: CATATGATGGTGCAGTGTCTCGAC<br>R: GAATTCGGTATCATCAACCTGCGA	Introducing *BdCBL3* to pGBKT7
BdCBL4-BD	F: GAATTCGGTATCATCAACCTGCGA<br>R: CCATGGAGCATCCTCAACCTGAGA	Introducing *BdCBL4* to pGBKT7
BdCBL5-BD	F: CATATGATGGTGGATTTTTTACGGCGGCTC<br>R: CCATGGAGCATCCTCGACCTGAGA	Introducing *BdCBL5* to pGBKT7
BdCBL7-BD	F: CATATGATGGGCTGTGCATCATCA<br>R: GGATCCCAACTCCTCGTCGCCAGA	Introducing *BdCBL7* to pGBKT7
BdCBL8-BD	F: GAATTCATGGCTTTGTTGGGTTCG<br>R: GGATCCGGTTTCCCTTAGTCTCGA	Introducing *BdCBL8* to pGBKT7
