# Supplementary material for: BdCIPK31, a Calcineurin B-Like Protein-Interacting Protein Kinase, Regulates Plant Response to Drought and Salt Stress
Source: Front Plant Sci. 2017 Jul 7;8:1184. doi: 10.3389/fpls.2017.01184 (PMC5500663; doi:10.3389/fpls.2017.01184)
Supplement: Supplementary file 2 [file Table_2.PDF]

Table S2. Primers used in RT and qRT PCR analyses

Gene	Sequence (5'-3')	Purpose
Bdactin	F: TGCCCAGCAATGTATGTCGC<br>R: CAGCAAGGTCCAGACGAAGG	Internal reference gene for *BdCIPK31* expression analysis
BdCIPK31	F: TTGTTTGGGTTTGTGAAG<br>R: AATGCTAGTTCCTGAGAA	*BdCIPK31* gene expression analysis
NtUbiquitin	F: TTGTGTCGTGTTGATTGA<br>R AAACAGCAAACAGAAATAGC	Internal reference gene for tobacco gene expression analysis
NtNCED1	F: AAGAATGGCTCCGCAAGTTA<br>R: GCCTAGCAATTCCAGAGTGG	*NtNCED1* gene expression analysis
NtABA2	F: GGCATAAGGTCTAAGGTA<br>R: TCTCAATATCAGCAGGAA	*NtABA2* gene expression analysis
NKT1	F: CCTAATGAATCTGATAGC<br>R: CGAGAAGTAGAAGAATAC	*NKT1* gene expression analysis
NKT2	F: CTCAAGGCAAGATTAGAT<br>R: TAAGAAGAACCATTACACA	*NKT2* gene expression analysis
NTKC1	F: GGACTTACTGCCTACTTA<br>R: TAAGAAGAACCATTACACA	*NTKC1* gene expression analysis
TORK1	F: CTTACAGAGATAGTCAGA<br>R: TGCTTAACCTAATCCATA	*TORK* gene expression analysis
NtSOS1	F: CAAATGTTATCCCCCGAAAGC<br>R: CGGAGAACCTGAGGAAATGTG	*NtSOS1* gene expression analysis
NtNHX2	F: ACTCATCCCCATTGGTCCG<br>R: AAGGAGTTCCACAAAAGCACGA	*NtNHX2* gene expression analysis
NtCAX3	F: CGGTTTGGCAATAATTGTCACAG<br>R: CAACGATCATGCTTCAATCATCC	*NtCAX3* gene expression analysis
NtCAT1	F: AGGTACCGCTCATTCACACC<br>R: AAGCAAGCTTTTGACCCAGA	*NtCAT* gene expression analysis
NtPOX2	F: ATAGGAACACTTATGAAG<br>R: GAGATAATGGTTGAGTTA	*NtPOX2* gene expression analysis
NtSOD	F: CTCCTACCGTCGCCAAAT<br>R: GCCCAACCAAGAGAACCC	*NtSOD* gene expression analysis
NtGST	F: AGCACCCTTACCTTTCCCTCA<br>R:GACATACTGGGCATCTTCTTTGG	*NtGST* gene expression analysis
NtDFR2	F: TATATGCTAAGAAGATGAC<br>R: GTATGATGCTAATGAAATC	*NtDFR2* gene expression analysis
NtAPX1	F: GACATTGCTATCAGACTC<br>R: CTCCAGTAACTTCAACAG	*NtAPX1* gene expression analysis
NtP5CS1	F: ATCTTCTAGTTCTGTTGA<br>R: CTCTCCTTAATGTATGTG	*NtP5CS1* gene expression analysis
NtSUS1	F: GTGGGGAAACACCGCTGAA<br>R: CAACAAGGATGCGAGGGATGA	*NtSUS1* gene expression analysis
NtADC	F: GCTGGATTGCCTTCAGTTGC	*NtADC* gene expression analysis

	R: TTCATACCCGCACCAAGACG	
NtSAMDC	F: CCATCCTAAAGTTGGCTGAGACC<br>R: ACTGAGCACCAGGGAAAATGAA	*NtSAMDC* gene expression analysis
NtABF1	F: ATAGCCTTACACTTGATGAG<br>R: CAACAGTCCATACAGTCTTA	*NtABF1* gene expression analysis
NtABF2	F: GCAGCCATCTATCTATTC<br>R: GCAACTCATCCATATTCA	*NtABF2* gene expression analysis
NtRD26	F: GCTCCCAAATGGGACGACCT<br>R: GCATTTGGGGCTCAAAAGGGT	*NtRD26* gene expression analysis
NtDREB3	F: GCCGGAATACACAGGAGAAG<br>R: CCAATTTGGGAACACTGAGG	*NtDREB3* gene expression analysis
NtERD10C	F: AACGTGGAGGCTACAGATCG<br>R: GTTCCTCTTGGGCATGAGTT	*NtERD10C* gene expression analysis
NtERD10D	F: GAGGACACGGCTGTACCAGT<br>R: GCGCCACTTCCTCTGTCTT	*NtERD10D* gene expression analysis
NtLEA5	F: TTGAATCTGGGGTTTTGGTT<br>R: GGAAGCATTGACGAGCTAGG	*NtLEA5* gene expression analysis
TobLTP1	F: GGTTTTGTGCATGGTGGTGG<br>R: CTTAGAGCAGTCTGTGGAGG	*TobLTP1* gene expression analysis
