# Supplementary material for: BdCIPK31, a Calcineurin B-Like Protein-Interacting Protein Kinase, Regulates Plant Response to Drought and Salt Stress
Source: Front Plant Sci. 2017 Jul 7;8:1184. doi: 10.3389/fpls.2017.01184 (PMC5500663; doi:10.3389/fpls.2017.01184)
Supplement: Supplementary file 3 [file Table_3.PDF]

Table S3. The *cis*-elements predicted in *BdCIPK31* promoter region.

***Cis*-elements**	**Position**	**Description**
ABRE	111<br>225<br>2018	AREB/ABF binding sites, involved in the abscisic acid responsiveness.
CAAT-motif	232<br>272<br>572	Light responsive element
G-Box	113<br>225<br>1045	Light responsive element
GARE	645<br>1448<br>1565	Gibberellin-responsive element
P box	1141<br>1865	Gibberellin-responsive element
LTR	941<br>106<br>1345<br>1698<br>1751	*Cis*-acting element involved in low-temperature responsiveness
Skn-1 motif	33<br>199<br>651	*Cis*-acting regulatory element required for endosperm expression
GCN4 motif	1102<br>1458	*Cis*-regulatory element involved in endosperm expression
Sp1	17<br>610<br>748<br>1161	Light responsive element
TC-rich repeats	396<br>1523	*Cis*-acting element involved in defense and stress responsiveness
TCA element	134<br>533<br>439<br>798<br>1112<br>1553	*Cis*-acting element involved in salicylic acid responsiveness
TGACG motif	130<br>705	*Cis*-acting regulatory element involved in the

		MeJA-responsiveness
5UTR Py-rich stretch	1113<br>1224<br>1549<br>1554	*Cis*-acting element conferring high transcription levels
MBS	1135<br>1652<br>1810	MYB binding site
TATA box	1690<br>1852<br>1931	Core promoter element around -30 of transcription start
