# Supplementary figures and images for: BdCIPK31, a Calcineurin B-Like Protein-Interacting Protein Kinase, Regulates Plant Response to Drought and Salt Stress
Source: Front Plant Sci. 2017 Jul 7;8:1184. doi: 10.3389/fpls.2017.01184 (PMC5500663; doi:10.3389/fpls.2017.01184)

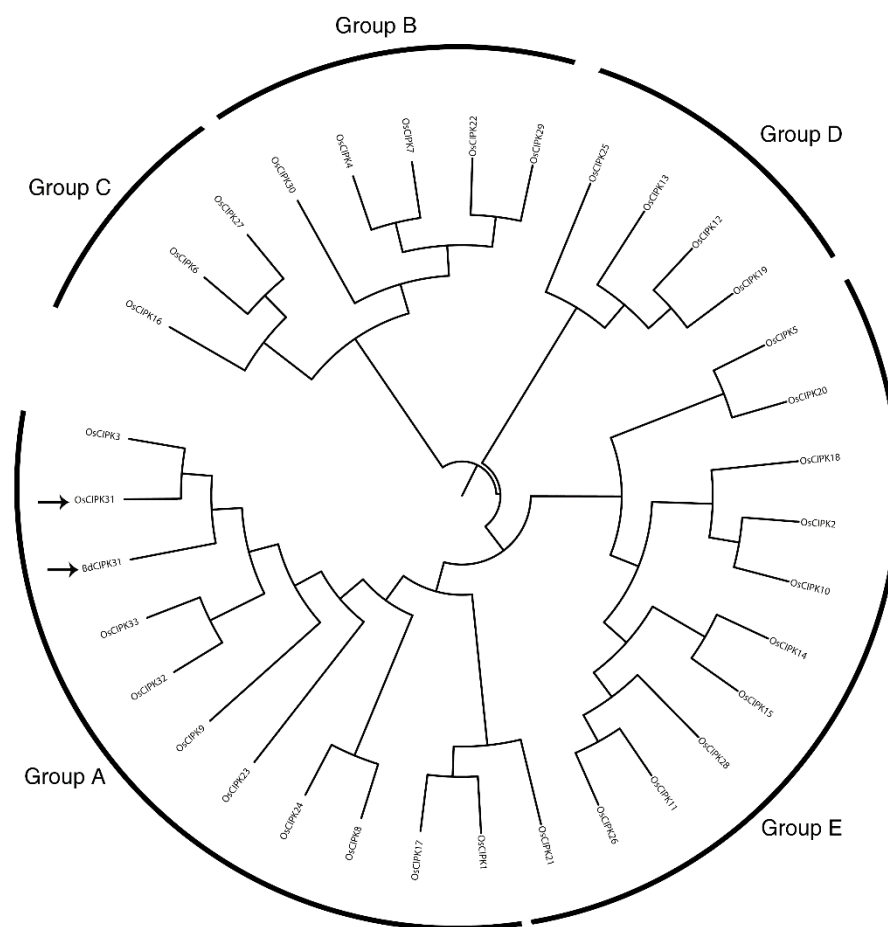

**Figure S1. Phylogenetic analysis of BdCIPK31 and OsCIPKs.** Arrows indicate BdCIPK31 and OsCIPK31.

Supplement: Supplementary file 4 [file Image_1.PDF]
