# Supplementary material for: BdCIPK31, a Calcineurin B-Like Protein-Interacting Protein Kinase, Regulates Plant Response to Drought and Salt Stress
Source: Front Plant Sci. 2017 Jul 7;8:1184. doi: 10.3389/fpls.2017.01184 (PMC5500663; doi:10.3389/fpls.2017.01184)
Supplement: Supplementary file 7 [file Image_4.PDF]

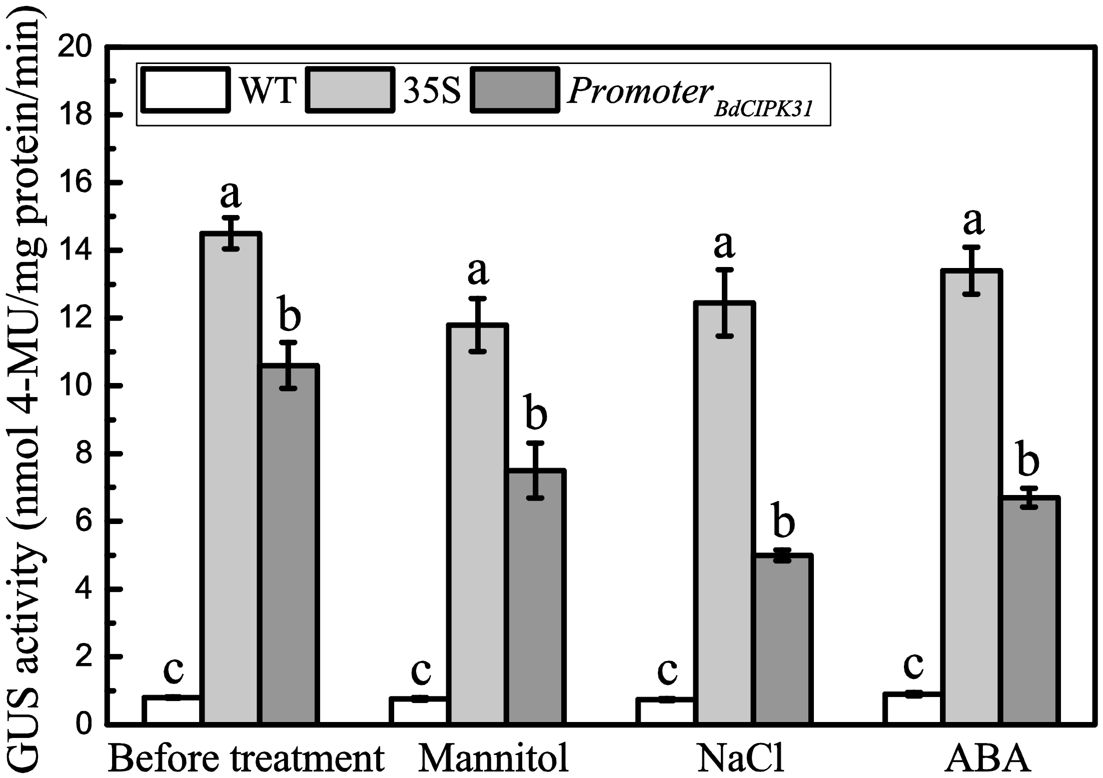

Figure S4. Quantitive GUS activity assay. *ProBdCIPK31:GUS* transgenic tobacco seedlings and control seedlings were subjected to 300 mM mannitol, 200 mM NaCl or 5 μM ABA treatment for 24 h and the GUS activity was detected by fluorometrical method. Data represent the means ± SE from three independent replicates. Different letters represent significant difference in each condition (Duncan's test, $P < 0.05$).
