# Supplementary material for: BdCIPK31, a Calcineurin B-Like Protein-Interacting Protein Kinase, Regulates Plant Response to Drought and Salt Stress
Source: Front Plant Sci. 2017 Jul 7;8:1184. doi: 10.3389/fpls.2017.01184 (PMC5500663; doi:10.3389/fpls.2017.01184)
Supplement: Supplementary file 8 [file Image_5.PDF]

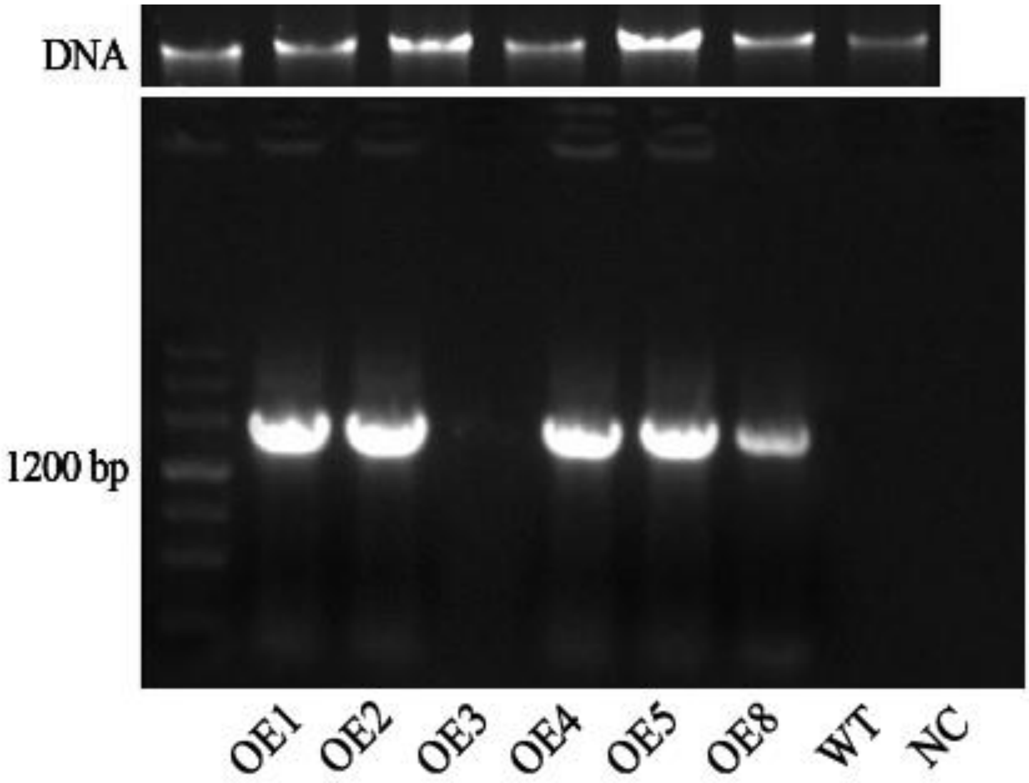

Figure. S5 Screening of transgenic tobacco lines. Transgenic tobacco lines overexpressing *BdCIPK31* were generated. DNA extracted from these lines (upper line) was subjected to PCR by using gene specific primers. Five independent transgenic lines were screened out. Wild type plant DNA and water were employed as negative and non-template control.
