# Supplementary material for: BdCIPK31, a Calcineurin B-Like Protein-Interacting Protein Kinase, Regulates Plant Response to Drought and Salt Stress
Source: Front Plant Sci. 2017 Jul 7;8:1184. doi: 10.3389/fpls.2017.01184 (PMC5500663; doi:10.3389/fpls.2017.01184)
Supplement: Supplementary file 9 [file Image_6.PDF]

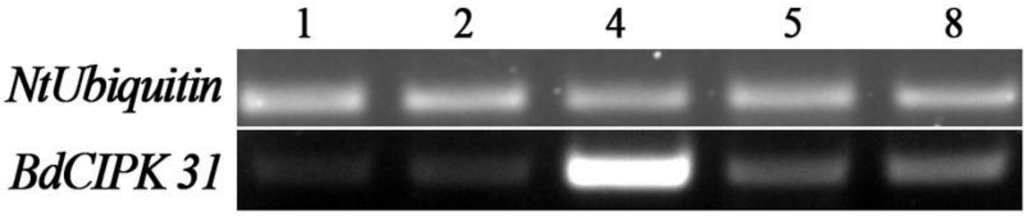

Figure S6. Analysis of the *BdCIPK31* gene expression in different transgenic tobacco lines. The expression level of *BdCIPK31* gene in different transgenic lines were examined by semi-quantitive RT-PCR.
