# Supplementary material for: BdCIPK31, a Calcineurin B-Like Protein-Interacting Protein Kinase, Regulates Plant Response to Drought and Salt Stress
Source: Front Plant Sci. 2017 Jul 7;8:1184. doi: 10.3389/fpls.2017.01184 (PMC5500663; doi:10.3389/fpls.2017.01184)
Supplement: Supplementary file 10 [file Image_7.PDF]

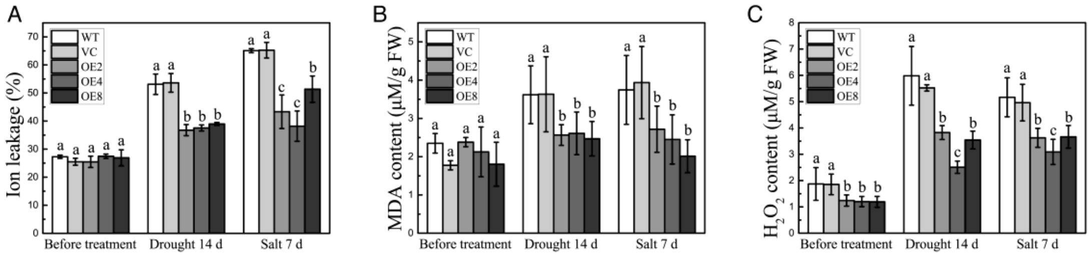

FigureS7. Analyses of ion leakage, MDA, and H_2O_2 content of the tobacco plants under drought or salt stress. (A) Ion leakage, (B) MDA content, (C) H_2O_2 content in the leaves of tobacco plants under drought or salt treatment. Data represent the means ± SE from three independent replicates. Different letters represent significant difference in each condition (Duncan's test, $P < 0.05$).
