# Supplementary material for: BdCIPK31, a Calcineurin B-Like Protein-Interacting Protein Kinase, Regulates Plant Response to Drought and Salt Stress
Source: Front Plant Sci. 2017 Jul 7;8:1184. doi: 10.3389/fpls.2017.01184 (PMC5500663; doi:10.3389/fpls.2017.01184)
Supplement: Supplementary file 11 [file Image_8.PDF]

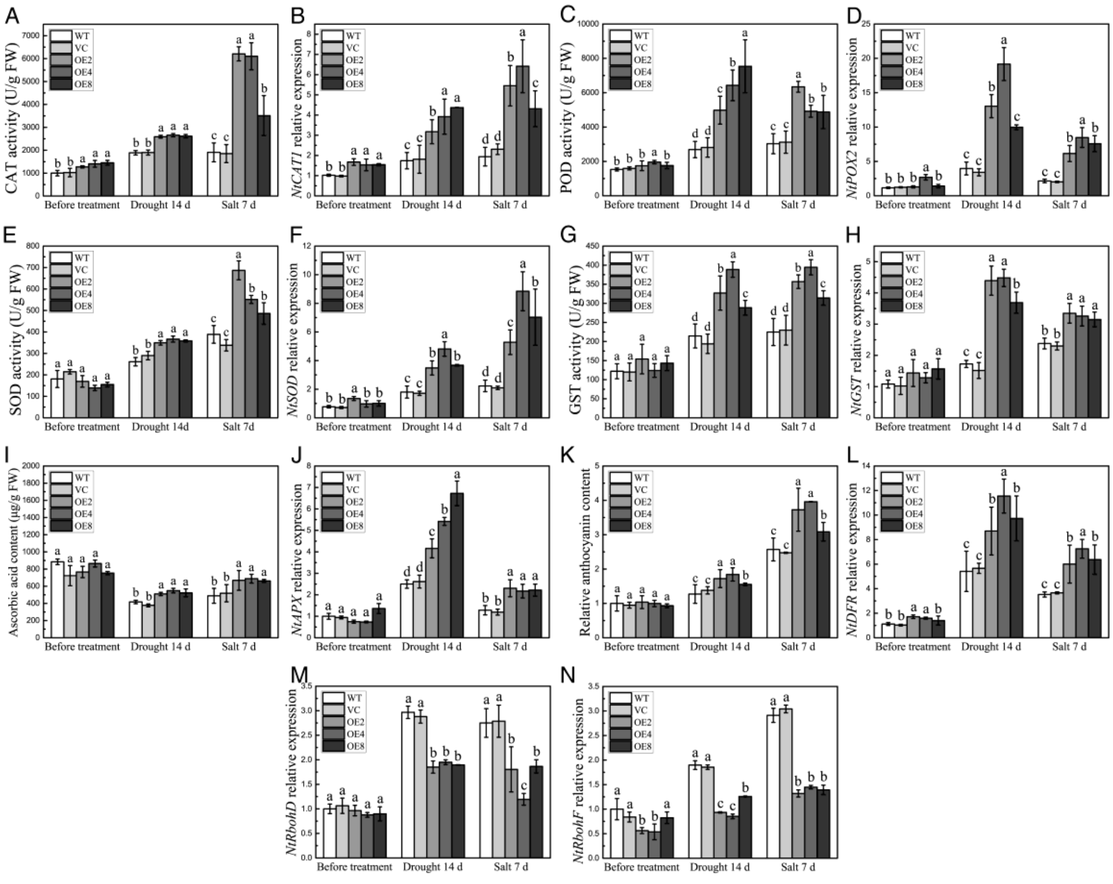

Figure S8. Overexpression of *BdCIPK31* affects ROS-scavenging system in transgenic tobacco under drought or salt stress. Analyses of the activity of (A) CAT, (C) POD, (E) SOD, and (G) GST activity, the content of (I) ascorbic acid and (K) anthocyanin, and the expression levels of (B) *NtCAT1*, (D) *NtPOX2*, (F) *NtSOD*, (H) *NtGST*, (J) *NtAPX*, (L) *NtDFR*, (M) *NtRbohD*, and (N) *NtRbohF*. Data represent the means ± SE from three independent replicates. Different letters represent significant difference in each condition (Duncan's test, $P < 0.05$).
