# Supplementary material for: BdCIPK31, a Calcineurin B-Like Protein-Interacting Protein Kinase, Regulates Plant Response to Drought and Salt Stress
Source: Front Plant Sci. 2017 Jul 7;8:1184. doi: 10.3389/fpls.2017.01184 (PMC5500663; doi:10.3389/fpls.2017.01184)
Supplement: Supplementary file 12 [file Image_9.PDF]

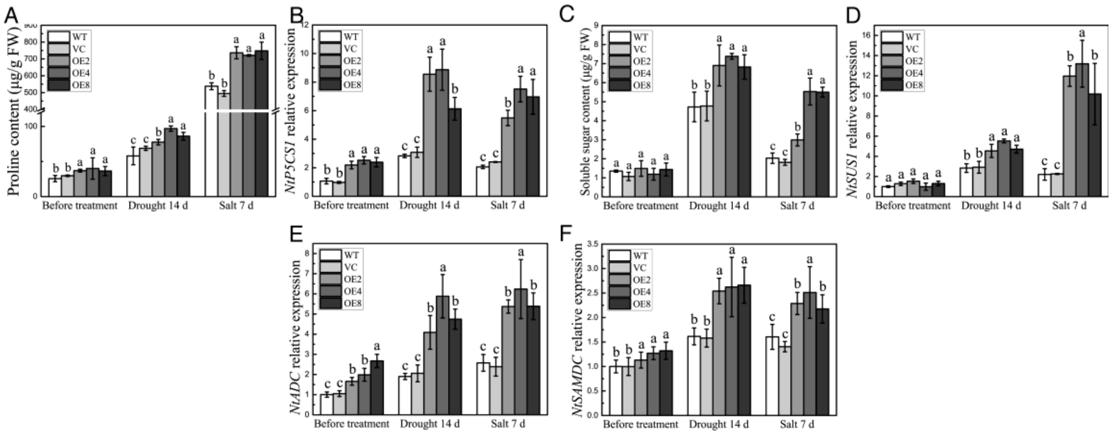

Figure S9. Overexpression of *BdCIPK31* affects osmolyte accumulation in transgenic tobacco plants. Analyses of the content of (A) proline and (B) soluble sugar, and the expression of (B) *NtP5CS1*, (D) *NtSUS1*, (E) *NtADC*, (F) *NtSAMDC* in the leaves of transgenic tobacco plants under drought or salt treatment. Data represent the means ± SE from three independent replicates. Different letters represent significant difference in each condition (Duncan's test, $P < 0.05$).
