# Supplementary material for: BdCIPK31, a Calcineurin B-Like Protein-Interacting Protein Kinase, Regulates Plant Response to Drought and Salt Stress
Source: Front Plant Sci. 2017 Jul 7;8:1184. doi: 10.3389/fpls.2017.01184 (PMC5500663; doi:10.3389/fpls.2017.01184)
Supplement: Supplementary file 13 [file Image_10.PDF]

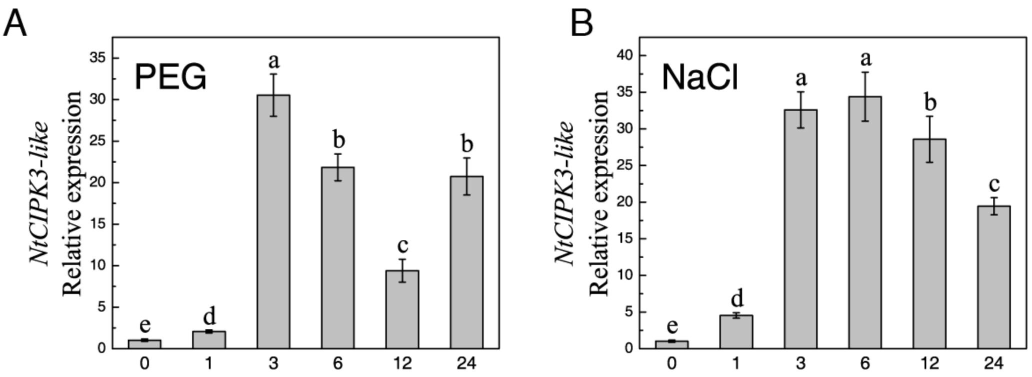

Figure S10. Expression of *NtCIPK3-like*, the homolog of *BdCIPK31* in tobacco, in response to PEG and NaCl treatment. 14-day-old tobacco seedlings were treated by (A) 20% PEG6000 and (B) 200 mM NaCl, respectively. The *NtCIPK3-like* expression in the shoots of treated seedlings were detected. Data represent the means ± SE from three independent replicates. Different letters represent significant difference in each condition (Duncan's test, $P< 0.05$).
