# Supplementary material for: BdCIPK31, a Calcineurin B-Like Protein-Interacting Protein Kinase, Regulates Plant Response to Drought and Salt Stress
Source: Front Plant Sci. 2017 Jul 7;8:1184. doi: 10.3389/fpls.2017.01184 (PMC5500663; doi:10.3389/fpls.2017.01184)
Supplement: Supplementary file 5 [file Image_2.PDF]

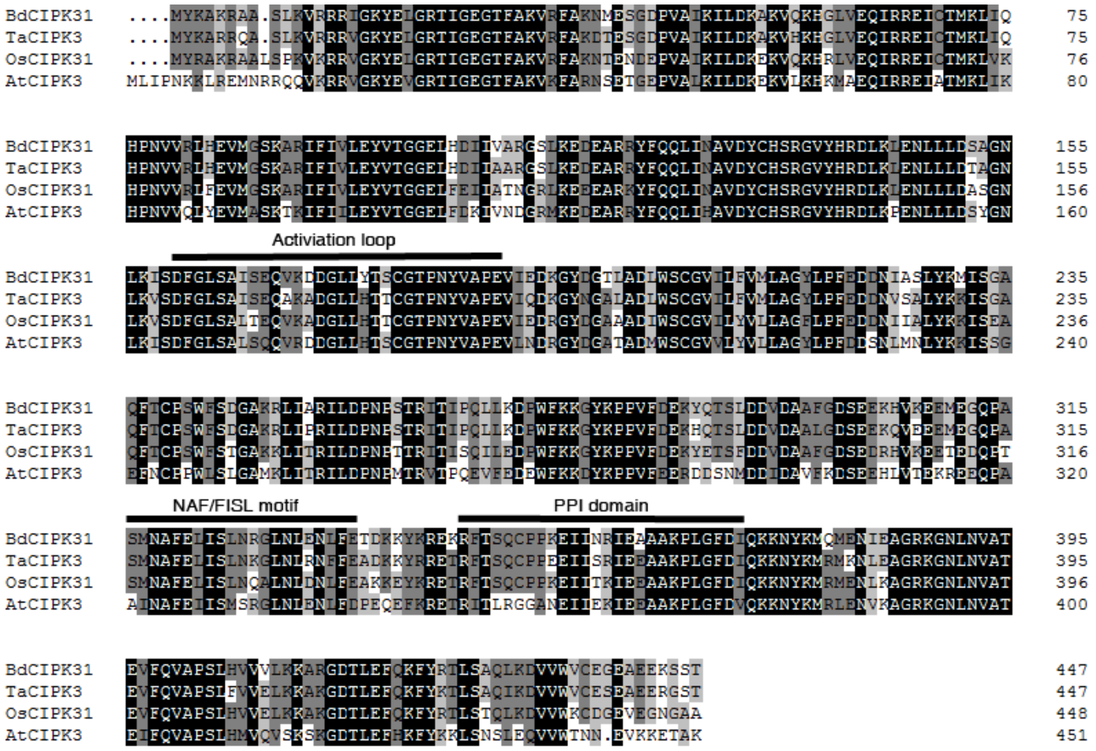

Figure S2. Multiple sequence alignment of BdCIPK31 and its homologs TaCIPK3, OsCIPK31 and AtCIPK3. Identical amino acid residues are shaded in black, similar amino acid residues are shaded in grey. Conservative motifs are indicated by line.
